# Identification, Characterization, and Expression Profile Analysis of the *mTERF* Gene Family and Its Role in the Response to Abiotic Stress in Barley (*Hordeum vulgare* L.)

**DOI:** 10.3389/fpls.2021.684619

**Published:** 2021-07-15

**Authors:** Tingting Li, Wenqiu Pan, Yiyuan Yuan, Ying Liu, Yihan Li, Xiaoyu Wu, Fei Wang, Licao Cui

**Affiliations:** ^1^College of Bioscience and Engineering, Jiangxi Agricultural University, Nanchang, China; ^2^State Key Laboratory of Crop Stress Biology in Arid Areas and College of Agronomy, Northwest A&F University, Yangling, China

**Keywords:** barley, *mTERF* gene family, duplication, expression profile, qRT-PCR, genetic variation

## Abstract

Plant mitochondrial transcription termination factor (mTERF) family regulates organellar gene expression (OGE) and is functionally characterized in diverse species. However, limited data are available about its functions in the agriculturally important cereal barley (*Hordeum vulgare* L.). In this study, we identified 60 *mTERF*s in the barley genome (*HvmTERF*s) through a comprehensive search against the most updated barley reference genome, Morex V2. Then, phylogenetic analysis categorized these genes into nine subfamilies, with approximately half of the *HvmTERF*s belonging to subfamily IX. Members within the same subfamily generally possessed conserved motif composition and exon-intron structure. Both segmental and tandem duplication contributed to the expansion of *HvmTERF*s, and the duplicated gene pairs were subjected to strong purifying selection. Expression analysis suggested that many *HvmTERF*s may play important roles in barley development (e.g., seedlings, leaves, and developing inflorescences) and abiotic stresses (e.g., cold, salt, and metal ion), and *HvmTERF21* and *HvmTERF23* were significant induced by various abiotic stresses and/or phytohormone treatment. Finally, the nucleotide diversity was decreased by only 4.5% for *HvmTERF*s during the process of barley domestication. Collectively, this is the first report to characterize *HvmTERF*s, which will not only provide important insights into further evolutionary studies but also contribute to a better understanding of the potential functions of *HvmTERF*s and ultimately will be useful in future gene functional studies.

## Introduction

One of the major differences between eukaryotes and prokaryotes is that the former has organelles, while the latter does not (Quesada, 2016). Due to endosymbiotic evolution from their cyanobacterial ancestors, most of the organellar genes within chloroplasts and mitochondria have been either lost or transferred to the nucleus (Gray, 2012). Current chloroplast and mitochondrial genomes retain only a tiny fraction of the genes, which are required for photosynthesis, gene expression, and electron transport chains (Lang et al., 1999; Yagi and Shiina, 2012; Robles and Quesada, 2021). Nevertheless, thousands of proteins have been predicted to be localized in plant mitochondria and chloroplasts according to bioinformatics analysis, most of which are encoded by the nuclear genome (Binder and Brennicke, 2003; Huang et al., 2013; Lee et al., 2013). Thus, the organellar gene expression (OGE) apparatus is a precisely coordinated system that largely depends on a great many of proteins encoded by nuclear genes (Pfannschmidt et al., 2015; Quesada, 2016). In the mitochondria and chloroplasts of higher plants, several transcriptional components of the functional OGE system have been reported. Three different polymerases involved in the transcriptional machinery have been demonstrated, including a multi-subunit plastid-encoded RNA polymerase (PEP) and two single-subunit nucleus-encoded RNA polymerases (NEPs) (Pfannschmidt et al., 2015). However, currently known auxiliary factors can only partially explain the transcriptional machinery, suggesting the existence of additional unidentified regulatory factors that are required for organellar gene transcription (Kühn et al., 2007; Liere et al., 2011).

Among the nucleus-encoded OGE factors, a novel protein family has received increasing concerns, namely, the mitochondrial transcription termination factor (mTERF) family. mTERF proteins, firstly characterized in animal mitochondria, were involved in mitochondrial transcription, translation, and DNA replication (Quesada, 2016). These proteins possess a variable number of ~30 amino acid “mTERF” motifs and comprise three leucine zipper-like elements separated by loops (Roberti et al., 2006), which are believed to confer the ability to recognize and bind to the typical mTERF motif on mitochondrial genome (Roberti et al., 2009). To date, four members, mTERF1 to mTERF4, have been described in vertebrates (Linder et al., 2005; Roberti et al., 2009). mTERF1, the founding member of this family, was originally considered to promote transcription termination of the heavy strand genes *tRNA-Ler* and *16S rRNA* (Kruse et al., 1989). However, more recent studies proposed that mTERF1 only partially terminated heavy strand transcription, whereas its major function was to completely block transcriptional interference at the opposite light strand of the ribosomal RNA genes from which they originated (Terzioglu et al., 2013). Although mTERF2 is a non-specific mitochondrial DNA binding protein and works as a negative regulator of mitochondrial gene expression, the function of mTERF2 is largely unknown (Pellegrini et al., 2009; Huang et al., 2011). Similarly, mTERF3 has been demonstrated as a specific repressor of mammalian mitochondrial transcription initiation, and therefore slowing down cell metabolism (Park et al., 2007). Meanwhile, other studies also revealed that mTERF3 was essential for ribosome biogenesis, mitochondrial protein transcription, and translation (Andersson et al., 2011; Wredenberg et al., 2013). mTERF4 can directly regulate mitochondrial ribosomal biogenesis and protein translation by targeting to the ribosomal RNA methyltransferase NSUN4 (a 5-methylcytosine RNA methyltransferase) (Cámara et al., 2011; Spåhr et al., 2012; Yakubovskaya et al., 2012).

By contrast, the *mTERF* gene family has expanded to approximately 30 members during the evolutionary process of land plants (Quesada, 2016; Leister and Kleine, 2020). For example, there are 35 *mTERF*s in *Arabidopsis thaliana*, 33 in rice (*Oryza sativa*) (Kleine, 2012), 31 in maize (*Zea mays*) (Zhao et al., 2014), 25 in grape (*Vitis vinifera*) (Yin et al., 2021), and 35 in *Capsicum annuum* (Tang et al., 2019). The substantial expansion in the number of *mTERF* genes was accompanied by their increased involvement in diverse RNA metabolism processes, with the majority being involved in rRNA maturation and intron splicing in organelles (Méteignier et al., 2020). For instance, *Arabidopsis mTERF8* mediates preferential transcription termination of the chloroplast gene *psbJ* by preferentially binding to the 3′-terminus (Xiong et al., 2020). *Arabidopsis* mTERF15 acts as an RNA binding protein that is required for mitochondrial *nad2* intron 3 splicing and functional complex I activity, which is indispensable for plant growth and development (Hsu et al., 2014). mTERF6 is required for the maturation of the chloroplast Ile transfer RNA gene *trnI.2* and regulates transcription termination of the PEP core subunit rpoA poly-cistron, thus further demonstrating the essential roles of mTERFs in leaf organogenesis and patterning in *Arabidopsis* (Romani et al., 2015; Robles et al., 2018b; Zhang et al., 2018). A more recent study reported that mTERF2 is implicated in the splicing of the group IIB introns of *ycf3* (intron 1) and *rps12* in *Arabidopsis*. Knock-down *mTERF2* resulted in delayed flowering time and knock-out *mTERF2* mutants were embryo lethal (Lee et al., 2021). In maize, ZmmTERF4 is involved in plastid ribosome accumulation and promote group II intron splicing of *trnI.2, trnA, rpl2, atpF*, and *ycf3-2* in chloroplasts (Hammani and Barkan, 2014). Zmsmk3 affects complex I assembly by modulating *nad4* intron 1 and *nad1* intron 4 splicing, seedling growth, and kernel development (Pan et al., 2019). Moreover, recent studies have also proposed the importance of *mTERF* genes associated with a variety of abiotic stress responses, including heat, salt, and osmotic stresses. For instance, *Arabidopsis* SHOT1 (SUPPRESSOR OF HOT1-4 1) can indirectly increase thermotolerance by reducing reactive oxygen species (ROS) accumulation and increasing the expression of heat shock proteins (HSPs), particularly those localized to mitochondria (Kim et al., 2012). mTERF5/MDA1 (mTERF DEFECTIVE IN *Arabidopsis*1) not only has a dual function in the transcription and stabilization of specific chloroplast transcripts but also responds to salt, osmotic, and sugar stresses through perturbed abscisic acid (ABA) retrograde signaling during seedling establishment in *Arabidopsis* (Robles et al., 2012; Ding et al., 2019; Méteignier et al., 2020). *Arabidopsis* mTERF9 regulates chloroplast gene expression and development and responds to sugar, ABA, salt, and osmotic stresses (Robles et al., 2015). Similar to *mTERF5* and *mTERF9*, loss of *Arabidopsis mTREF27* resulted in mitochondria developmental defects and altered response to salt stress (Jiang et al., 2021). *Arabidopsis* mTERF10 and mTERF11 are involved in the response to salt stress, possibly through the ABA-mediated pathway (Xu et al., 2017). Recently, a new role was demonstrated for mTERF6 in response to adverse environmental stresses, such as ABA, ionic, and osmotic stresses (Robles et al., 2018a). *Arabidopsis* SOLDAT10 (SINGLET OXYGEN-LINKED DEATH ACTIVATOR10) controls plastid-specific rRNA expression and protein synthesis in plastids and is well known for its roles in the response to mild photooxidative stress (Meskauskiene et al., 2009). Collectively, *mTERF*s are essential for the regulation of OGE and play crucial roles in plant growth and development and in response to diverse abiotic stresses, at least in *Arabidopsis* and possibly in other higher plants. Nevertheless, detailed information about the molecular mechanisms of *mTERF*s is still rather limited in diverse plants, especially crop plants (Zhao et al., 2014).

As one of the earliest domesticated crops of ancient civilizations, barley (*Hordeum vulgare* L.) currently ranks as the fourth most abundant crop in terms of both area and tonnage harvested (Mayer et al., 2012). Barley is more adaptable to a wide range of agroclimatic conditions than its relative wheat and, as a result, is of high importance for human food, animal feed, and malt brewing (Jayakodi et al., 2020). The first draft sequence assembly of barley (Mayer et al., 2012) and its subsequent improved versions (Mascher et al., 2017; Monat et al., 2019) lay the foundation for the comprehensive identification and characterization of gene families at the genome-wide level. Here, the protein sequences of barley mTERFs were identified through a comprehensive search. The physicochemical properties, phylogenetic relationships, exon-intron gene structure, conserved motifs, expression profiles, and preliminary functions were systematically analyzed. Moreover, the single-nucleotide polymorphism (SNP) variation atlas of *mTERF*s for wild and landrace barley accessions was profiled. This study will not only shed light on the evolutionary mechanism of barley *mTERF*s, but also pave the way for their functional characterization in barley and beyond.

## Materials and Methods

### Identification of *mTERF* Gene Family Members in Barley

The protein sequences of barley Morex V2 were downloaded from the IPK database (https://doi.org/10.5447/ipk/2019/8), and the hidden Markov model (HMM) file of the mTERF domain (PF02536) was retrieved from the Pfam database. HMMER v2.41.1 was employed to search for the mTERF domain against the barley genome with the default inclusion threshold. The candidate sequences were further confirmed by using the NCBI-CDD (National Coalition Building Institute, Conserved Domains Database) (https://www.ncbi.nlm.nih.gov/cdd/), SMART (Simple Modular Architecture Research Tool) (http://smart.embl-heidelberg.de/), HMMER (https://www.ebi.ac.uk/Tools/hmmer/), and InterPro (http://www.ebi.ac.uk/interpro/search/sequence/) online tools. Subsequently, a BLAST (Basic Local Alignment Search Tool) search against barley ESTs (Expressed Sequence Tag) was conducted to determine the existence of putative *mTERF* genes. The molecular weight (MW), number of amino acids, theoretical isoelectric point (pI), and grand average of hydropathicity (GRAVY) were evaluated using the online tool ExPASy (http://web.expasy.org/protparam/). The subcellular localization was predicted using the TargetP online tools (http://www.cbs.dtu.dk/services/TargetP/).

### Phylogenetic Relationship, Gene Structure, and Conserved Motif Analysis

Multiple sequence alignment of full-length proteins of *HvmTERF* genes was performed using the Clustal X program. An unrooted neighbor-joining (NJ) phylogenetic tree was constructed using MEGA X with 1,000 bootstrap replicates. The exon-intron gene structure was visualized using the Gene Structure Display Sever (GSDS) (http://gsds.cbi.pku.edu.cn/) based on the gene annotation GTF (Gene Transfer Format) file. The conserved protein motifs were obtained using online MEME (Multiple Em for Motif Elicitation) tools (https://meme-suite.org/meme/) with the following parameters: the maximum number of motifs was set to 10, any number of repetitions was allowed, and the optimum width ranged from 6 to 250. The 1.5 kb genomic sequences upstream of the coding regions were extracted and submitted to the PlantCARE database (http://bioinformatics.psb.ugent.be/webtools/plantcare/html/) to identify the putative *cis*-acting regulatory elements in the promoter region. The transcripts of *HvmTERF*s were extracted and submitted to the psRNATarget online server (http://plantgrn.noble.org/psRNATarget/) to detect the candidate miRNA targets with the following parameters: published miRNAs from *Brachypodium*, barley, and wheat were chosen, with a maximum expectation = 4.

### Gene Duplication and Comparative Genomics Analysis of Barley, *Arabidopsis, Brachypodium*, Rice, Grape, and Maize *mTERF*s

To reveal the duplication events of *HvmTERF* during barley evolution, an integrated method was employed to identify the duplicated pairs. First, MCScanX software was used to detect duplication events. Second, the following criteria were used as described by Chen et al. (1) the alignment of shorter genes covered ≥70% of longer genes; (2) the aligned region possessed an identity ≥70%; and (3) only one duplication event was counted for tightly linked genes (Gu et al., 2002; Chen et al., 2012). The duplicated events were manually combined into a non-redundant dataset to determine the orthologous relationships between barley and other species. The orthologs of *mTERF* genes in *A. thaliana, Brachypodium distachyon, O. sativa, V. vinifera*, and *Z. mays* were identified using InParanoid V4.1. The syntenic blocks within and among species were detected by MCscanX. To evaluate the evolutionary rate of the duplicated and syntenic genes, PAML (Phylogenetic Analysis by Maximum Likelihood) v4.3 software was utilized to calculate the non-synonymous (Ka) and synonymous (Ks) substitution ratios. The duplicated pairs were visualized using Circos v0.67 software.

### Expression Analysis of *HvmTERF* Genes

To estimate the gene expression profile of *HvmTERFs*, RNA-seq samples from different tissues and developmental stages as well as plants responding to various biotic and abiotic stresses were retrieved from the NCBI Sequence Read Archive (SRA) database (https://www.ncbi.nlm.nih.gov/). The sample information and accession numbers are shown in Supplementary Table 1. The Hisat2 v2.1.0 and StringTie v1.3.5 pipelines were employed to calculate the fragments per kilobase of transcript per million fragments mapped (FPKM) value. The R package Ballgown was used to identify the differentially expressed genes. The differentially expressed genes were identified as having a false discovery rate (FDR) ≤ 0.05 and fold change ≥2. The heatmap and hierarchical clustering were generated using the pheatmap package embedded in R with the log2 transformed FPKM values. To determine the co-expressed genes with *HvmTERF*s, a co-expression network was constructed by weighted gene co-expression network analysis (WGCNA) in R. Here, a convenient one-step method was employed for network construction, and genes with the top 5% weighted values associated with *HvmTERF*s were categorized for further analysis. A BLAST search against *Arabidopsis* and rice proteins was performed to determine the potential functions of the co-expressed genes. Cytoscape v3.8.0 was implemented to display the co-expression networks.

### Plant Materials, Treatment, and qRT-PCR Analysis

Seeds of the barley cultivar Morex were sterilized with 5% sodium hypochlorite for 10 min, rinsed with distilled water, and then germinated on wet filter paper at 25°C for 5 days. The germinated seeds were hydroponically cultured in a greenhouse under the following conditions: 20°C day/15°C night, 16 h light/8 h dark cycle, and 50% relative humidity. Three-leaf-stage seedlings were exposed to 150 mM NaCl, 20% PEG, 4°C, or 100 μM ABA for 0, 1, 6, 12, 24, and 48 h. Seedlings without any treatment at the same time point were used as the control. Leaves and roots were collected from three plants at each time point and promptly frozen in liquid nitrogen for RNA extraction with three biological replicates.

To further investigate the possible functions of *HvmTERF*s, a total of 25 *HvmTERF*s were randomly selected to detect their expression patterns through qRT-PCR (Quantitative Real-time PCR) analysis. The primers used in this study are listed in Supplementary Table 2. Total RNA was isolated using a Plant RNA extraction kit (Omega Biotek, USA), and cDNA was synthesized using 5X All-in-one RT MasterMix (ABM, Canada) following the manufacturer's instructions. *HvACTIN2* (GenBank accession no. AY145451.1) was used as the internal control. The TB-Green® Premix Ex Taq™ II kit (Takara, Dalian, China) was used for qRT-PCR amplification in a QuantStudio™ Real-Time PCR system (Thermo Fisher, USA). The reaction protocol was as follows: 95°C for 30 s, followed by 40 cycles at 95°C for 3 s and 60°C for 30 s. The relative expression levels of candidate genes were calculated using the 2^−ΔΔCT^ method. Three technical replicates were applied for each treatment (Livak and Schmittgen, 2001). Student's *t*-test was employed for statistical analysis by R software. The histogram was drawn using the ggplot package in R software. One asterisk (^*^) and double asterisk (^**^) indicate 0.05 and 0.01 significance level, respectively.

### Nucleotide Variation, Population Structure, and Haplotype Analysis of *HvmTERF*s

To acquire the candidate *HvmTERFs*, a total of 220 barley resequencing samples were downloaded from the SRA database (Russell et al., 2016). The geographic distribution is presented in Supplementary Figure 1. The detailed material information is listed in Supplementary Table 3. BWA-MEM v0.7.13r1126 was used to map the clean reads against the barley reference genome. The PICARD-GATK pipeline was employed to generate single nucleotide polymorphisms (SNPs) with default parameters. The genomic distribution and potential function of the SNPs were annotated by SnpEFF v4.3. The SNPs located within the *HvmTERF* genes were retained for subsequent analysis. To further reveal the population structure of barley samples based on *HvmTERF* sequences, population structure analysis, phylogenetic tree analysis, and principal component analysis (PCA) were performed. ADMIXTURE v1.3.0 was used to infer the population structure with predefined K-values ranging from 2 to 5. The phylogenetic tree was constructed using Treebest v1.9.2. The Smartpca toolkit implemented in EIGENSOFT v4.2 was employed to conduct the PCA. Median-joining haplotype networks were constructed using the software programs DnaSP v5.10.01, Alignment v1.3.1.1, and Network v4.6.1.1. The network was visualized using Cytoscape v3.8.0. The nucleotide diversity (π) and Wright's F-statistic (Fst) were calculated using vcftools v0.1.16.

## Results

### Identification of *mTERF* Gene Family Members in Barley

The updated reference genome of barley, Morex v2, provided invaluable resources for *HvmTERF* identification, and a total of 60 *mTERF* genes were identified in barley using a combined method (Supplementary Tables 4, 5). Since there was no standard nomenclature, the barley *mTERF*s were designated as *HvmTERF1* to *HvmTERF60* according to their chromosome numbers and physical positions. The physicochemical properties of the HvmTERFs were further characterized. In detail, the *mTERF*s encoded proteins ranging from 105 (HvmTERF41) to 632 (HvmTERF59) amino acids in length, with pIs ranging from 5.41 (HvmTERF2) to 10.78 (HvmTERF10), and MWs ranging from 12.01 (HvmTERF41) to 71.82 kDa (HvmTERF59). The GRAVY values ranged from 0.259 (HvmTERF25) to −0.502 (HvmTERF7), with an average of −1.005. Most (61.67%) of the HvmTERFs displayed positive GRAVY values, suggesting hydrophobic characteristics. Subcellular location prediction revealed that most (78.33%) HvmTERFs were localized to mitochondria (39 HvmTERF*s*, 65%) or chloroplasts (8 HvmTERF*s*, 13.3%), and the remaining 13 HvmTERFs were targeted to other locations. To confirm the existence of *HvmTERF*s, a BLAST search against barley ESTs was performed. In total, 48 members of the *HvmTERF* gene family had EST records, whereas the remaining 12 *mTERF*s had no EST support, suggesting their stage- or tissue-specific expression profile or undetectable expression level.

### Phylogenetic and Structural Domain Analysis of HvmTERFs

We examined the amino acid sequence features of the mTERF domain by multiple sequence alignment. The conserved mTERF motifs spanned approximately 30 amino acids in length, have been characterized in other plants and are believed to act as DNA-binding modules (Zhao et al., 2014) (Supplementary Figure 2; Supplementary Table 6). The sequence conservation percentages for each amino acid residue were calculated, and 15 amino acid sites were highly conserved with a consensus sequence percentage >60%. Consistent with previous studies (Zhao et al., 2014), three repeats of the leucine zipper-like heptad X_3_LX_3_ were identified in barley mTERF motifs, of which the conservation percentages were 62.71, 46.67, and 36.67% for Leu-8, Leu-16, and Leu-23, respectively. These results revealed that HvmTERFs possessed well-characterized mTERF motifs with conserved leucine residues like those in other plants, indicating the conserved evolutionary process of plant mTERF proteins. Surprisingly, the conservation percentages of Ile-1 and Tyr-20 in barley were significantly higher than those in *Arabidopsis*, rice, and maize, suggesting that these residues may play essential roles in the evolutionary history of *HvmTERFs*.

**Figure 1:**
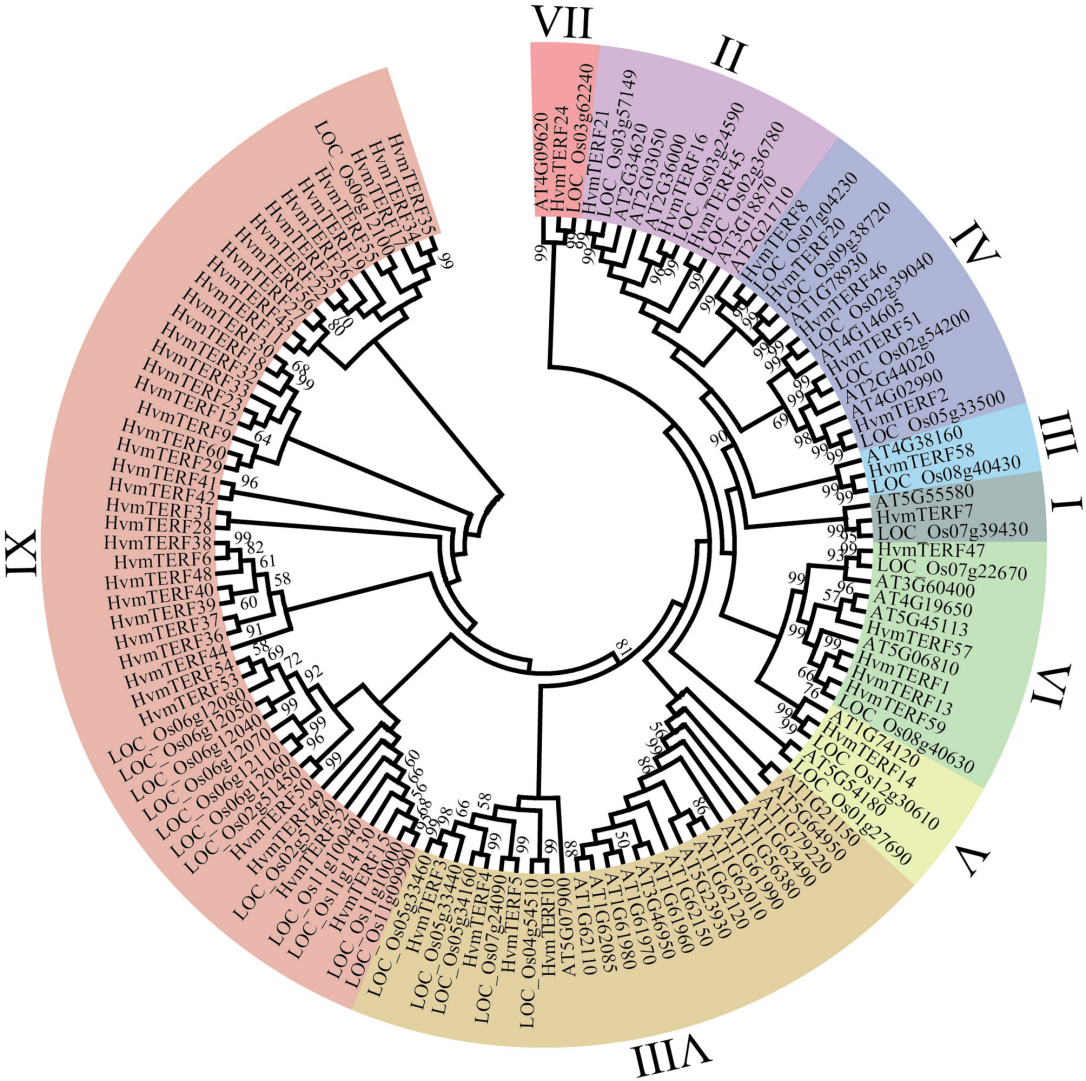
Phylogenetic analysis of mTERF proteins from *Arabidopsis*, rice, and barley. The phylogenetic tree was constructed using the Neighbor-joining method with 1,000 bootstrap replications. The nine subfamilies are marked with different colors.

To further elucidate the evolutionary relationship of *HvmTERF*s, we constructed a phylogenetic tree based on the alignment of 128 mTERF protein sequences from *Arabidopsis* (35), rice (33), and barley (60) (Figure 1). These mTERF proteins were divided into nine monophyletic clades according to the classification given by Zhao (Zhao et al., 2014). The number of proteins assigned to different subfamilies varied greatly, of which subfamily IX contained 39 members, whereas subfamilies I, III, V, and VII possessed only one member.

**Figure 2:**
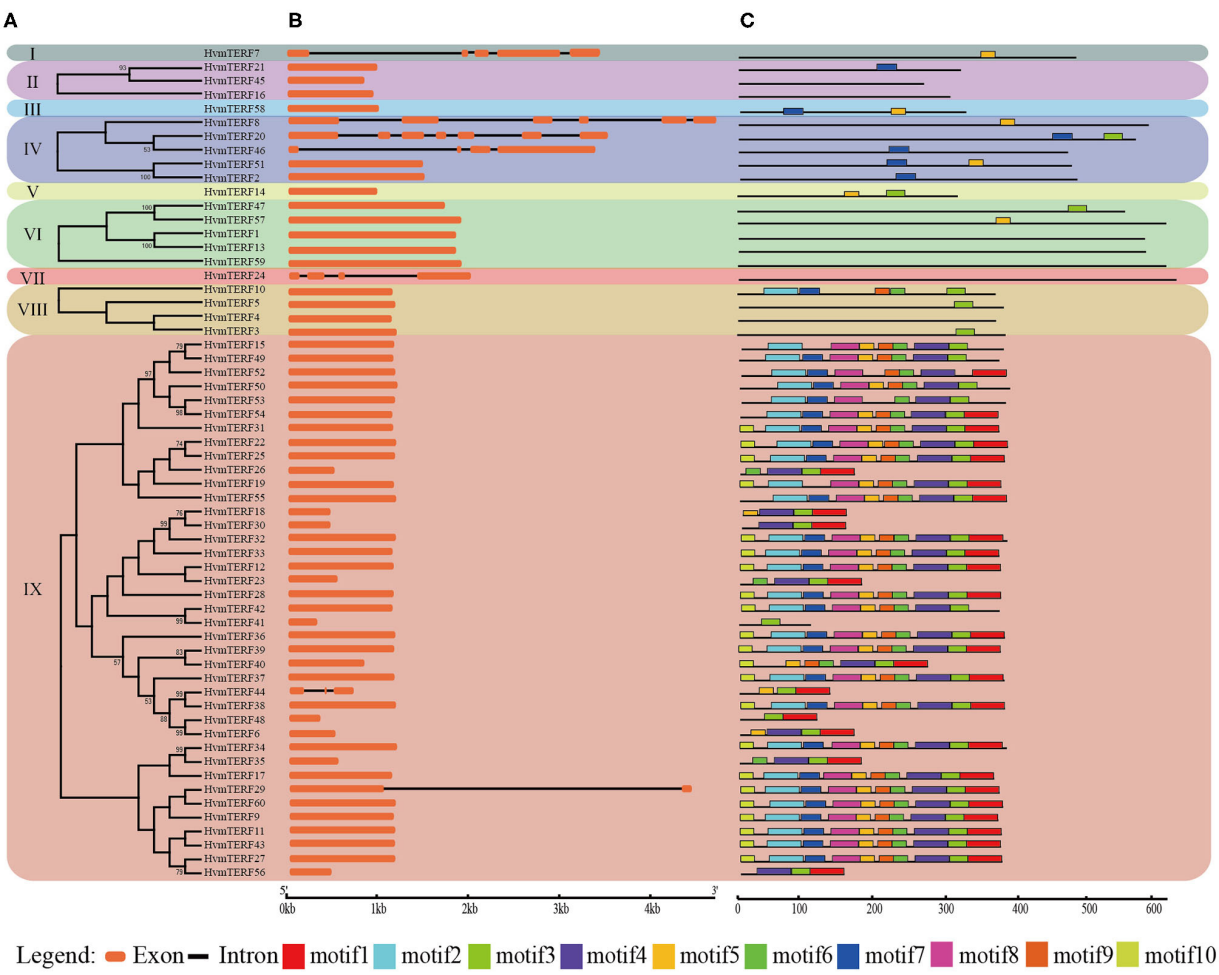
Phylogenetic tree, conserved motifs, and exon-intron structure analysis of *HvmTERF*s. **(A)** Phylogenetic tree for each subfamily. **(B)** Gene structure of *HvmTERF* genes. Exons are indicated as orange boxes. Black lines represent introns. **(C)** The motif composition of *HvmTERF*s. Motifs are designated as 1–10 and represented by different colors.

The exon–intron structure not only provides additional evidence to support the phylogenetic topology but also increases the understanding of the functional diversification within a gene family. Therefore, the exon-intron structure of *HvmTERF*s was analyzed to obtain their evolutionary relationships (Figure 2). A solid correlation between gene structures and their phylogeny was observed. Genes clustered within the same subfamily displayed a similar exon-intron structure. Indeed, *HvmTERF*s within subfamilies II, VI, and VIII showed nearly identical exon lengths and tended to be intron-less. Nonetheless, we pinpointed the exon/intron gain/loss event within several clusters. For example, 37 out of 39 *HvmTERF*s within subfamily IX possessed only 1 exon, whereas *HvmTERF29* and *HvmTERF44* had 2 and 3 exons, indicating that they may have acquired additional exons during the evolutionary history of the *mTERF* gene family.

To further gain insight into the evolutionary relationships and functional regions among the HvmTERFs, the distribution pattern of the conserved motifs was also visualized (Figure 2). We identified 10 motifs and designated them as motif 1 to motif 10. Notably, no motif was identified within HvmTERF*s 1, 4, 13, 16, 24, 45*, and *59*, possibly because the consensus sequence failed to reach the threshold in the MEME software. The HvmTERFs within the same clade showed similar motif numbers and distribution patterns. Motif 3 was shared by 44 members, ranking as the most abundant motif, followed by motif 4 (36) and motif 5 (36). Except for motifs 2, 5, 6, 7, and 9, the remaining motifs were specific to subfamily IX. We also observed a certain order of the identified motifs. For example, motif 2 tended to be tightly connect with motif 7, motif 5 was linked to motif 8, motif 6 was linked to motif 9, and motifs 1, 3, and 4 were linked.

### Duplication Events and Orthologous Analysis of *HvmTERF*s

The *HvmTERF*s were unevenly located across the seven barley chromosomes in accordance with the barley genome annotation, of which 27 *HvmTERF*s were located on chromosome 6, ranking as the most populated chromosome, whereas the other six chromosomes had only 9 (chromosome 7H) to 3 (chromosome 3H) *HvmTERF* genes (Supplementary Figure 3). Interestingly, there was no positive correlation between chromosome length and the number of *HvmTERF*s (Pearson correlation *r* = −0.2994, *p*-value = 0.5141), indicating that longer chromosomes do not necessarily contain more *HvmTERF* genes.

**Figure 3:**
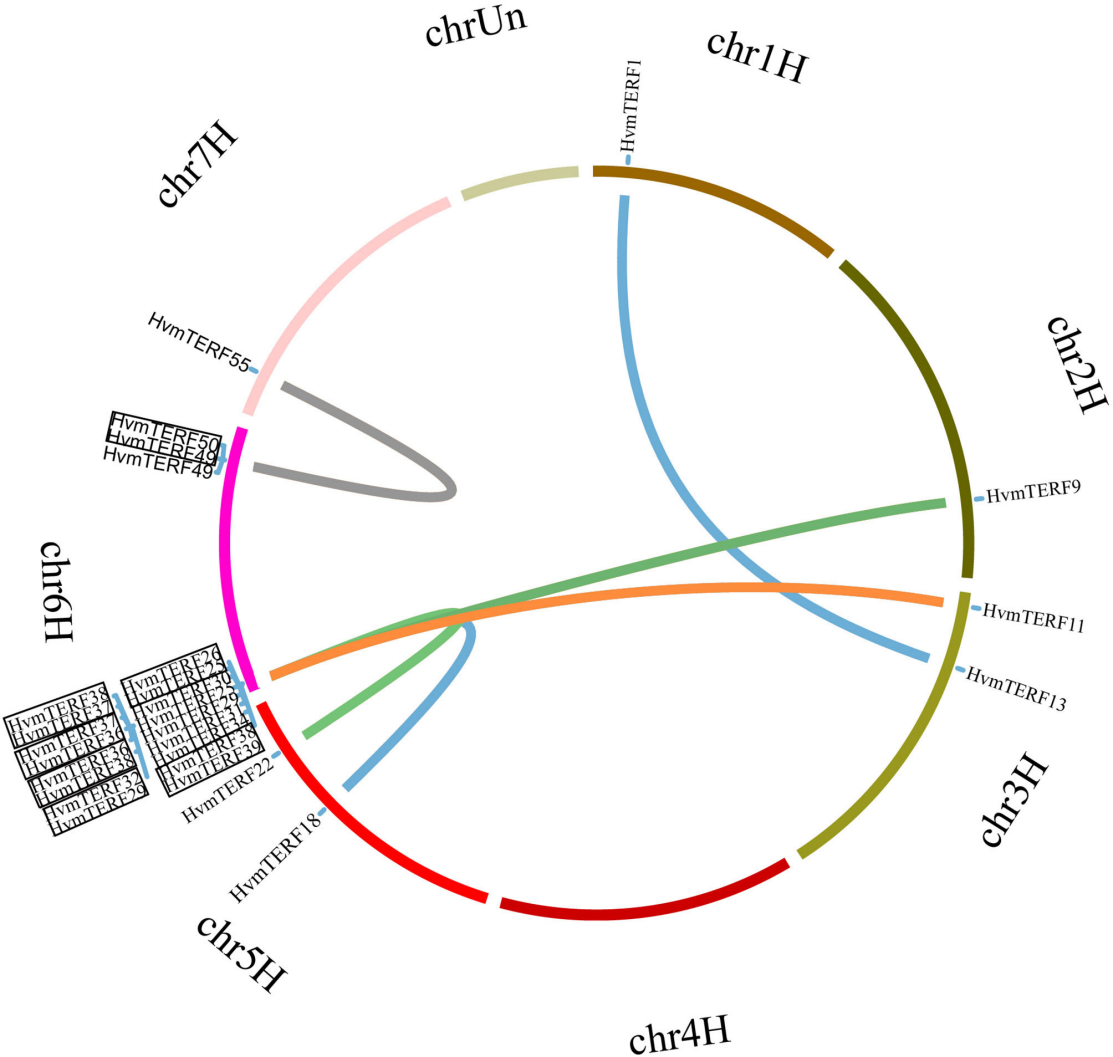
Chromosomal location and gene duplication of *HvmTERF*s. Tandemly duplicated gene pairs are highlighted with black boxes.

In order to elucidate the expansion mechanism of *HvmTERF*s, tandem, and segmental duplication event analyses were performed using an integrated method (Figure 3). The results showed that 10 *HvmTERF*s (*HvmTERFs 25* and *26, HvmTERFs 29* and *32, HvmTERFs 49* and *50*, and *HvmTERFs 36, 37, 38*, and *39*) were clustered into four tandemly duplicated regions on chromosome 6, indicating a gene distribution hot spot of *HvmTERF*s. It is noteworthy that all tandemly duplicated genes belonged to subfamily IX. Generally, it is difficult to segregate this kind of tightly linked gene arrangement through recombination in breeding or research. Furthermore, seven duplicated pairs composed of 13 *HvmTERF* genes were identified as segmental duplications. Except for *HvmTERF1* and *HvmTERF13*, the remaining duplicated genes were clustered in subfamily IX. Remarkably, six segmentally duplicated gene pairs were associated with chromosome 6. Taken together, both tandem and segmental duplication events contributed to the expansion of *HvmTERF*s, mainly in subfamily IX.

The non-synonymous (Ka) vs. synonymous (Ks) substitution ratios for duplicated gene pairs were further calculated to estimate the evolutionary constraints acting on *HvmTERF*s. Ka/Ks >1, =1, and <1 indicate positive, neutral and purifying selection, respectively (Lynch and Conery, 2000). In this study, the Ka/Ks ratio for duplicated gene pairs ranged from 0.1516 to 0.5662, with an average value of 0.3845, suggesting that these gene pairs have experienced strong purifying selective pressure during their expansion process (Supplementary Table 7).

**Figure 4:**
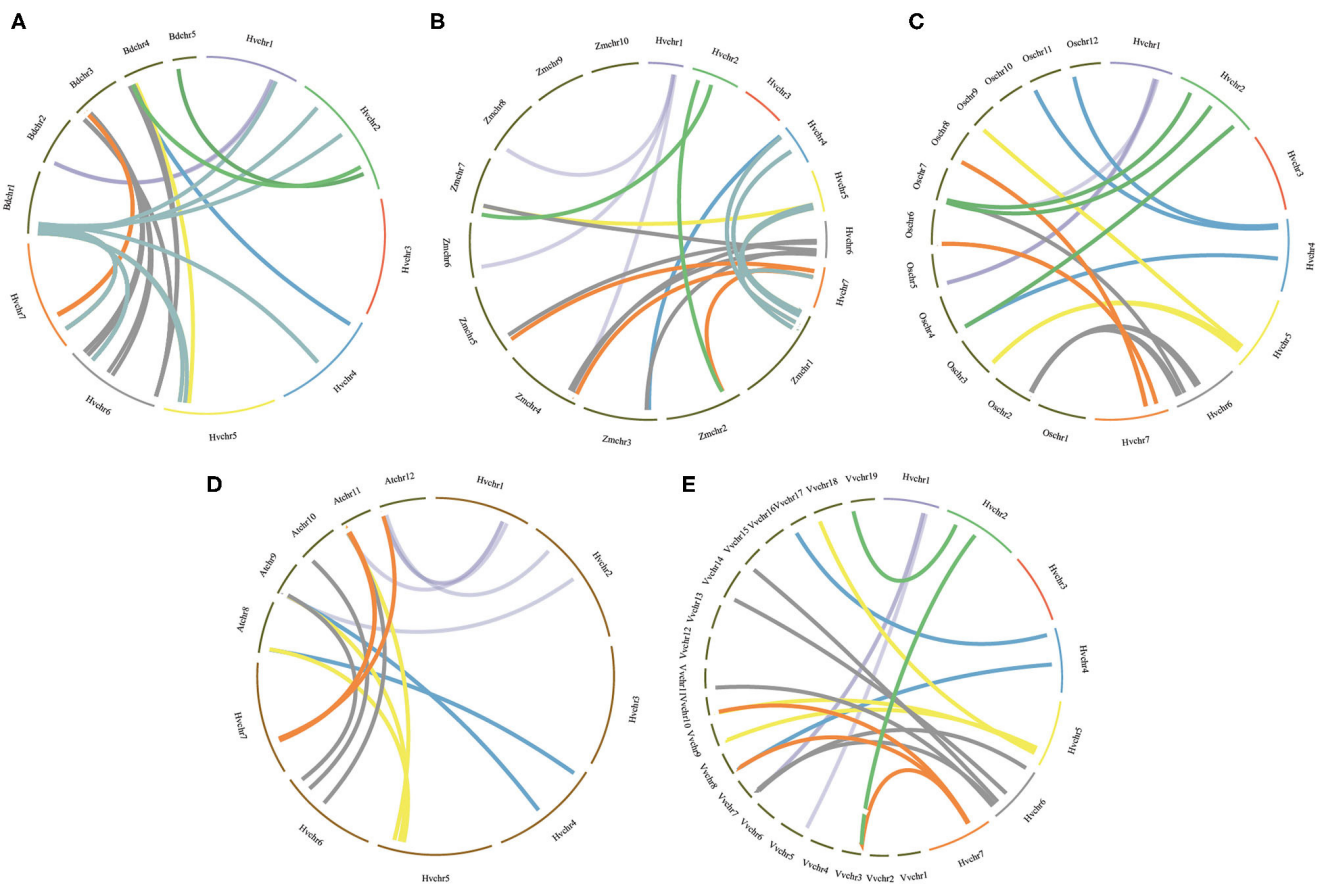
Orthologous analysis of *HvmTERF*s between barley and other five representative plant species. **(A)** *Brachypodium distachyon*, **(B)** *Zea mays*, **(C)** *Oryza sativa*, **(D)** *Arabidopsis thaliana*, **(E)** *Vitis vinifera*.

To further infer the evolutionary mechanisms of *HvmTERF*s, we conducted comparative ortholog analysis with five representative species, including two dicots (*A. thaliana* and *V. vinifera*) and three monocots (*B. distachyon, O. sativa*, and *Z. mays*) (Figure 4). The ortholog analysis resulted in 16, 18, 39, 22, and 20 gene pairs between barley and the other five species (*A. thaliana, V. vinifera, B. distachyon, O. sativa*, and *Z. mays*), respectively. A total of 29 *HvmTERF* genes held orthologous relationships with those in *B. distachyon*, followed by *O. sativa* (22), *Z. mays* (19), *V. vinifera* (18), and *A. thaliana* (16). Nine *HvmTERF*s (*HvmTERF*s *2, 3, 5, 8, 14, 16, 46, 58*, and *59*) were found to possess one-to-one relationships among the five representative species. We thus proposed that these evolutionarily conserved genes may have essential roles during plant evolution. Interestingly, 21 gene pairs composed of 12 *HvmTERF*s were only identified between barley and *B. distachyon, O. sativa*, and *Z. mays*, suggesting that these orthologous pairs formed after the divergence between monocotyledonous and dicotyledonous plants. The Ka/Ks ratios of the *mTERF* gene pairs were also calculated. All orthologous *mTERF* gene pairs showed Ka/Ks <1, suggesting that these *HvmTERF*s might have been subjected to extensive purifying selective pressure (Supplementary Table 8).

### Analysis of *cis*-elements and miRNA Target Sites of *HvmTERFs*

*Cis*-elements play vital roles in the transcriptional regulation of genes during plant growth and development as well as the plant response to biotic and abiotic stresses. By searching the PlantCARE database, a total of 40 *cis*-elements were identified and further classified into four categories. As shown in Supplementary Table 9, a total of 12 kinds of light-responsive elements were observed, accounting for the majority of the putative *cis*-elements. There were 10 hormone-responsive regulatory elements present in the *HvmTERF* promoters, such as ABRE (ABA-Responsive Elements), CGTCA-motif/TGACG-motif, TGA-element, ERE (Estrogen Response Element), and TCA-element, which were associated with ABA, methyl MeJA (Methyl Jasmonate), auxin, ethylene, and SA (Salicylic Acid), respectively. We also identified several organogenesis-related *cis*-elements, such as MSA-like (Mitosis-Specific Activator) (cell cycle regulation, 4 genes), GCN4 (General Control Non-repressible-4) (endosperm expression, 3 genes), CAT-box (meristem expression, 32 genes), AC-I/II (xylem expression, 2 genes), and circadian (circadian control, 11 genes). Notably, five kinds of biotic and abiotic stress-related regulatory elements, including MBS (Myeloblastosis Binding Site), GC-motif, ARE (Anaerobic Response Element), LTR (Long Terminal Repeat), and Wun-motif, which responded to drought, anoxic-specific inducibility, anaerobic induction, low temperature, and wound damage, respectively, were identified in the *HvmTERF* promoter regions. Therefore, the variety and quantity of regulatory elements were present in distinct *HvmTERF* promoters, suggesting their potential functions in diverse signal transduction pathways and various stress adaptations in barley.

To obtain preliminary insight into the miRNA-mediated posttranscriptional regulatory mechanisms, the CDSs (Coding Sequence) of *HvmTERF*s were extracted to search for miRNA target sites. The results showed that a total of 12 mTERF-miRNA pairs were identified, referring to five miRNAs targeting 10 *HvmTERF*s (Supplementary Table 10). Most of the miRNAs controlled the expression of *HvmTERF*s by guiding mRNA cleavage, whereas *HvmTERF34* and *HvmTERF35* were regulated by translation inhibition. *HvmTERF22* and *HvmTERF46* were targeted by miRNA6192 upstream of the mTERF domain, whereas a total of six *HvmTERF*s were targeted by miRNA9662a-3p within the mTERF domain. miRNA7717b-5p and miRNA9962a-3p both targeted *HvmTERF19* through transcript cleavage. Our findings suggested that miRNA was involved in the posttranscriptional regulation of *HvmTERF*, but the actual regulatory mechanism should be validated in future molecular biology experiments.

### Expression Profile Analysis of *HvmTERF* Genes

**Figure 5:**
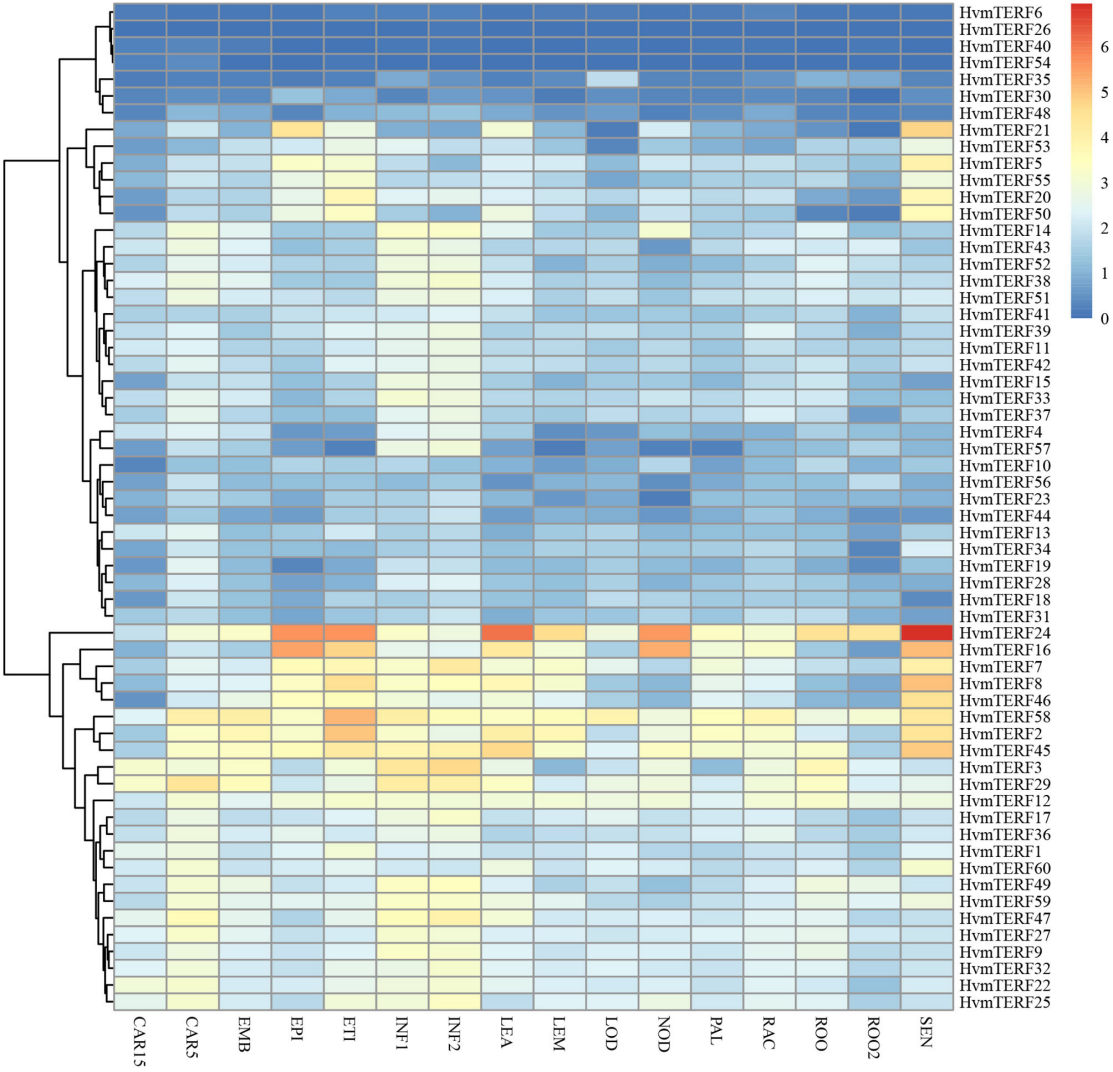
The expression profile of *HvmTERF* genes at different tissues or stage of barley. FPKM values were normalized by log_2_(FPKM+1) transform to represent color scores. CAR15/CAR5, developing grain (15/5 day after pollination); EMB, embryonic tissue (4 days); EPI, epidermal strips (4 weeks after pollination); ETI, etiolated seedling with 10 days old after planting; INF1, Young developing inflorescences with 5 mm; INF2, developing inflorescences with 1 cm; LEA, 10 cm shoots from seedlings; LEM, inflorescences, lemma(6 weeks after pollination); LOD, inflorescences, lodicule (6 weeks after pollination); NOD, developing tillers at third stem internode (6 weeks after pollination); PAL, dissected inflorescences, palea (6 weeks after pollination); RAC, inflorescences, rachis (5 weeks after pollination); ROO, roots from the seedlings with 17 and 28 days old after planting; ROO2, roots (4 weeks after pollination); SEN, senescing leaves (8 weeks after pollination).

To obtain preliminary insight into tissue- and stage-specific expression profiles and elucidate the potential roles of *mTERF*s in tissue development, the transcript abundances of *HvmTERF*s in 16 different tissues or stages were obtained using Illumina RNA-seq data. As shown in Figure 5, *HvmTERF*s were expressed in all barley RNA-seq samples studied. The *mTERF*s were highly expressed in seedlings, leaves and developing inflorescences. *HvmTERFs 2, 7, 8, 16, 24, 45, 46*, and *58* exhibited relatively high expression levels in most of the studied tissues and stages, suggesting these genes may play an important role in barley growth and development. It is noteworthy that *HvmTERF24* ranked the most highly expressed gene with an average FPKM value of 28.58. We also identified tissue- and stage-specific *HvmTERF*s. *HvmTERF3, HvmTERF5, HvmTERF30, HvmTERF35*, and *HvmTERF50* exhibited preferential expression in young inflorescences, senescing leaves, epidermis, lodicules, and senescing leaves, respectively. *HvmTERF20* was predominantly expressed in seedlings and senescing leaves, whereas *HvmTERF21* was mostly expressed in the epidermis and senescing leaves, suggesting that these genes were involved in tissue- or stage-specific development in barley. Interestingly, four *HvmTERF* genes (*HvmTERFs 6, 26, 40*, and *54*) in subfamily IX exhibited almost no expression in any of the tissues and stages.

**Figure 6:**
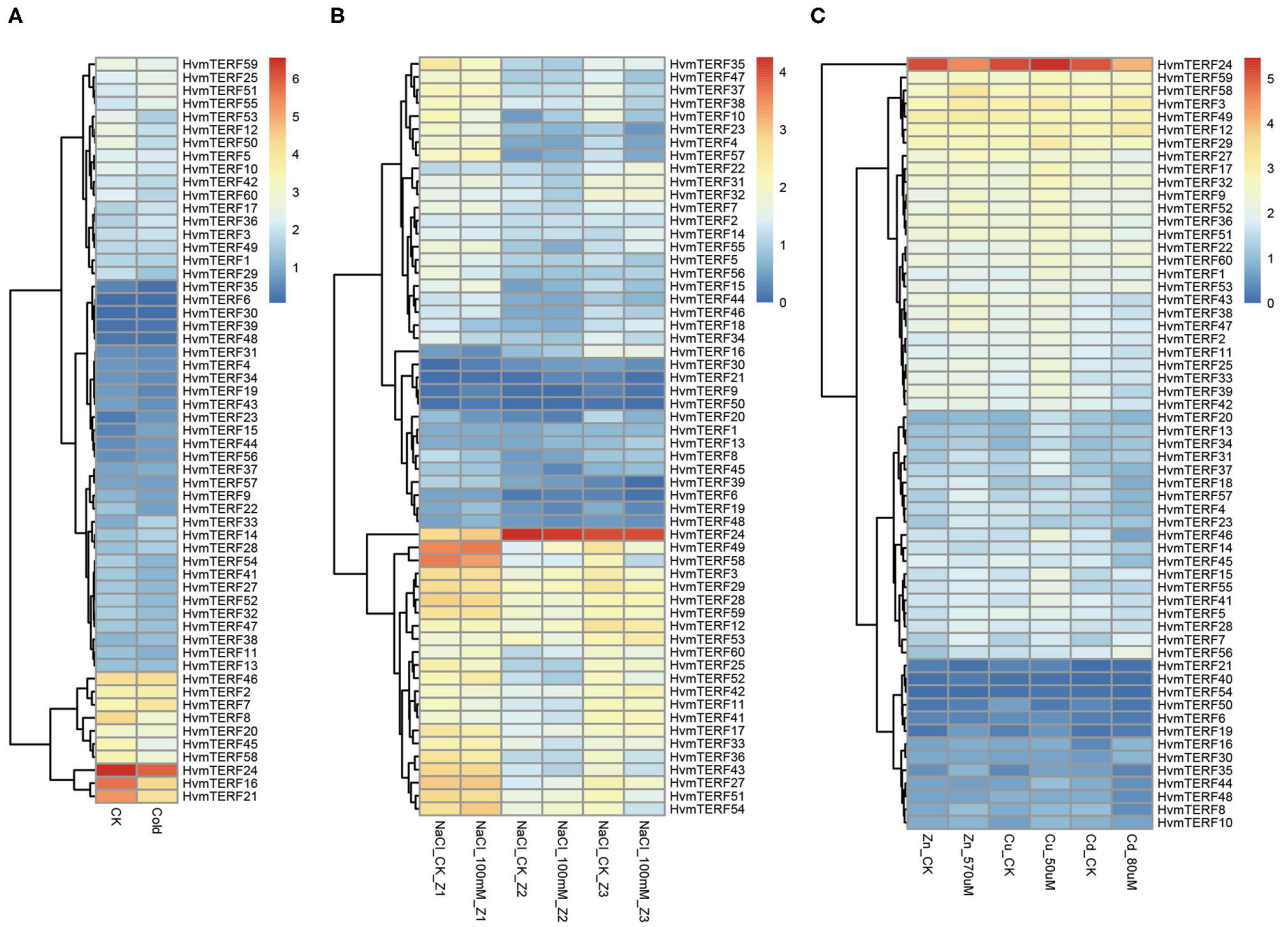
Expression profiles of *mTERF* genes under five stress conditions. **(A)** cold stress; **(B)** salt stress; **(C)** zinc, copper, and cadmium stress.

To gain comprehensive information about the functions of *HvmTERF*s in response to abiotic stresses, the expression profiles of *HvmTERFs* under cold, salt, and metal ion stresses were further investigated. The results revealed that *HvmTERFs 15, 23*, and *33* were found to be upregulated under cold treatment, whereas seven *HvmTERF* genes were downregulated (Figure 6A). Notably, the tissue-specific gene *HvmTERF16* (*mTERF3/SL1, SEEDLING LETHAL 1*) was downregulated with 2.65-fold change compared with the control. Since limited studies have been conducted on *mTERF* genes (Jiang et al., 2020), the biological function of *HvmTERF*s still need more experimental verification.

The expression patterns of *HvmTERFs* under salt treatment were further analyzed. A total of 5, 2, and 15 *HvmTERF* genes were differentially expressed in the root meristematic zone, elongation zone, and maturation zone, respectively (Figure 6B). Consistent with the expression pattern under cold treatment, most (72.72%) of the differentially expressed genes, including 1 in the meristematic zone and 15 in the maturation zone, were downregulated. Furthermore, *HvmTERF50* was upregulated in the meristematic zone and downregulated in the mature zone. *HvmTERF10* was upregulated in the elongation zone and downregulated in the mature zone. Notably, *HvmTERF21* was upregulated in both the meristematic zone (3.49-fold) and elongation zone (4.01-fold) and downregulated in the maturation zone (2.95-fold).

We finally analyzed the expression profiles of *HvmTERFs* under metal ion toxicity stress. Among them, 4, 5, and 2 upregulated *HvmTERF*s were found to be zinc-, copper- and cadmium toxicity-responsive genes, and 2, 1, and 9 downregulated genes were also identified (Figure 6C). Remarkably, the expression levels of *HvmTERF19* under zinc and copper treatment were 4.14- and 2.45-fold higher than those of the control. *HvmTERF35* was significantly upregulated under zinc and copper treatment and downregulated under cadmium treatment. However, *HvmTERF50* was upregulated under zinc treatment and downregulated under copper and cadmium treatment. *HvmTERF21* was upregulated under cadmium treatment and downregulated under zinc treatment.

### Co-expression Network Analysis Between *HvmTERF*s and Other Genes in Barley

**Figure 7:**
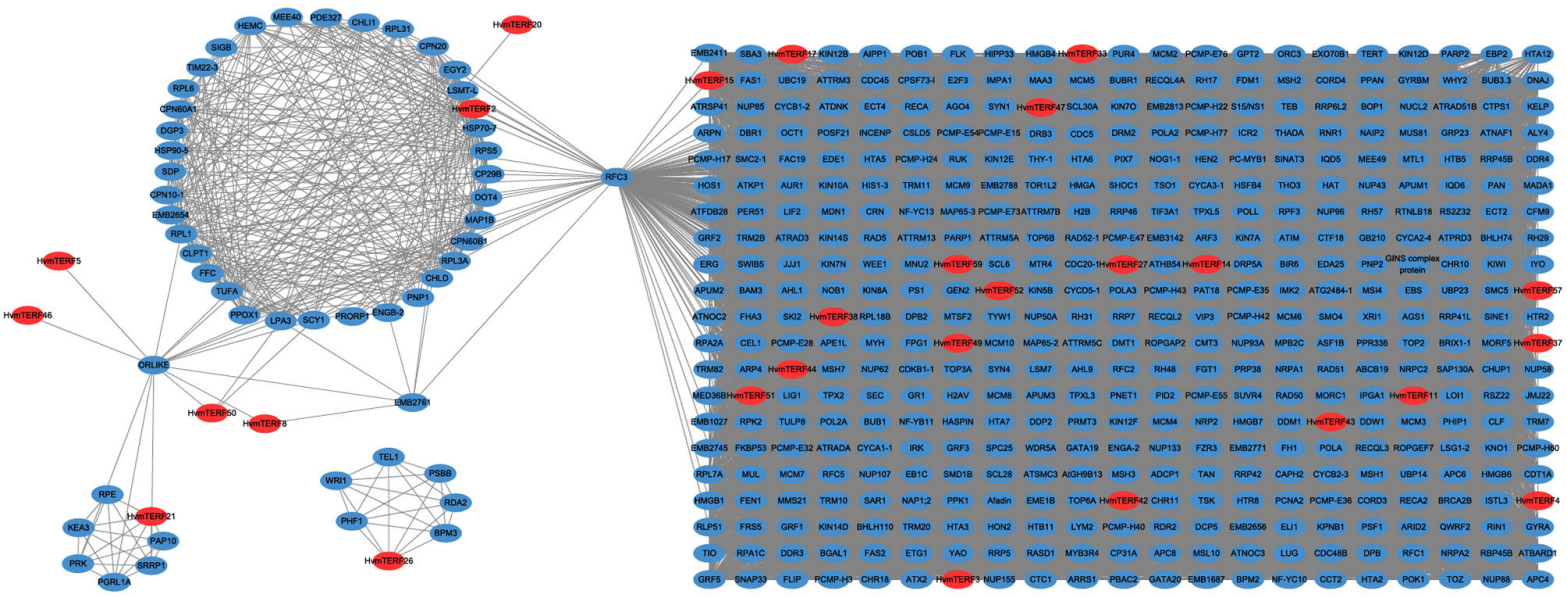
The co-expression network analysis of *HvmTERFs* with other barley genes. Only annotated genes are represented.

Co-expression analysis has become an effective methodology for gene functional annotation (Wei and Chen, 2018). By using a large dataset of 148 RNA-seq samples, we attempted to construct a co-expression network of *mTERF* genes. A total of 162,373 interactions, composed of 27 *HvmTERF*s and 778 other co-expressed genes, were detected (Figure 7). In detail, 595 (76.48%), 260 (33.42%), 178 (22.88%), and 167 (21.47%) genes were predicted to be co-expressed with *HvmTERF57, HvmTERF3, HvmTERF15*, and *HvmTERF33*, respectively. The results suggested that these *HvmTERF*s may play central regulatory roles in the co-expression network. Interestingly, nine *HvmTERF*s were co-expressed with multiple transcription factors. For instance, 5 *B3*, 4 *GRF* (*Growth-Regulating Factor*), 3 *C3H* (*Cysteine3Histidine*), and 3 *MYB* (*Myeloblastosis*) family genes were co-expressed with 4, 4, 7, and 3 *HvmTERF*s. Transcription factors are essential regulators to repress or activate the expression of their target genes by binding to specific upstream elements (Jin et al., 2017). These results suggested that multiple transcription factors may interact with *HvmTERF*s, and further to control multitudinous growth and development processes, and response to environmental stressors in barley. Furthermore, 11 *HvmTERF*s were predicted to be co-expressed with *SPLICEOSOME-ASSOCIATED PROTEIN 130* (*SAP130a*), a key gene that is required for specific spatiotemporal events during reproduction in *Arabidopsis* (Aki et al., 2011).

Gene Ontology (GO) enrichment analysis was further performed to determine the potential function of the *mTERF* co-expressed genes. The *mTERF* co-expressed genes were enriched in 155 GO terms (FDR <0.05), including 66 biological processes, 41 cellular components, and 48 molecular functions (Supplementary Figures 4–6; Supplementary Table 11). In the molecular function category, microtubule binding (GO:0008017), nucleoside-triphosphatase activity (GO:0017111), and tubulin binding (GO:0015631) ranked as the top three enriched terms, whereas intracellular non-membrane-bound organelle (GO:0043232), non-membrane-bound organelle (GO:0043228), and nucleus (GO:0005634) were the most common terms in the cellular components category. In the biological process category, the *mTERF* co-expressed genes were implicated not only in various biological functions (e.g., GO:0006260 DNA replication metabolic process; GO:0006259 DNA metabolic process; GO:0042254 ribosome biogenesis) but also in response to diverse stresses (e.g., GO:0033554 cellular response to stress; GO:0006974 cellular response to DNA damage stimulus).

### Expression of *HvmTERF* Genes in Response to Salt, Drought, Cold, and ABA Treatment via qRT-PCR

Although differentially expressed *HvmTERF*s under different stresses were obtained based on RNA-seq data, comprehensive expression patterns of *HvmTERF*s in response to various stresses and phytohormones have not been reported. To better understand the expression patterns in response to diverse stress treatments (cold, salt, heat, and ABA), 25 *HvmTERF*s were randomly selected for qRT-PCR analysis. Under salt stress, all the candidate *HvmTERF*s were downregulated after 1, 3, and 12 h of treatment. *HvmTERF*23 was the most downregulated gene at the 1 h (5.93-fold), 3 h (12.89-fold), and 12 h (6.63-fold) time points (Supplementary Figure 7). Under 6 h salt treatment, nine *HvmTERF*s were found to be upregulated. Among them, the expression level of *HvmTERF*46 was dramatically increased with a 2.28-fold change value. Moreover, three upregulated *HvmTERF* genes were found at 24 h. Notably, *HvmTERF21* exhibited significantly upregulated expression levels at 6 and 24 h.

Under drought treatment, a total of 1, 1, 5, and 5 *HvmTERF* genes were upregulated at 3, 6, 12, and 24 h (Supplementary Figure 8). Among them, *HvmTERF*21 showed 1.21-, 1.36-, 1.24-, and 2.58-fold changes at the 3, 6, 12, and 24 h time points, whereas *HvmTERF*52 displayed 1.99- and 2.00-fold changes at the 12 and 24 h time points, respectively. The MBS *cis*-acting element involved in drought inducibility was also detected within the promoter regions of these genes (Urao et al., 1993). For example, *HvmTERFs52* possessed 2 MBS *cis*-acting elements. There were some exceptions, however, no MBS-acting element was detected in the promoter regions of *HvmTERF21*, implying this gene may have unknown elements acting in response to drought stress. Furthermore, *HvmTERFs* 2, 8, 19, 29, 43, and 49 were downregulated after drought treatment at all-time points.

Under cold treatment, we identified more upregulated *HvmTERFs* than those identified in response to salt and drought treatment, with 17, 19, 18, 17, and 20 upregulated genes after 1, 3, 6, 12, and 24 h of treatment, respectively (Supplementary Figure 9). Notably, *HvmTERFs 9, 16, 21, 24, 25, 45, 49*, and *51* were upregulated at all treated time points. The expression level of *HvmTERF*s dramatically decreased over time. The average fold change was 4.40 after 1 h of cold treatment, whereas it decreased to 1.24 after 48 h of stress, suggesting that *HvmTERFs* may mainly function in the initial response to cold injury.

The plant hormone ABA has been demonstrated to play important roles in improving the tolerance of plants to diverse stresses (Shinozaki and Yamaguchi-Shinozaki, 1997). qRT-PCR was also carried out to analyze the expression profiles of the 25 selected *HvmTERFs* after ABA treatment (Supplementary Figure 10). The heatmap revealed that *HvmTERFs 9, 16, 21, 24, 28*, and *29* exhibited upregulated expression patterns at all time points. Meanwhile, abundant ABRE *cis*-acting elements, the major *cis*-element for ABA-responsive gene expression, were identified in the promoter regions, such as five ABRE *cis*-element for *HvmTERF28*, three for *HvmTERF16*, and three for *HvmTERF24*. The expression level of *HvmTERF46* displayed upregulated expression with a 10.16-fold change after 6 h of treatment, whereas *HvmTERF53* showed 3.89- and 3.84-fold changes after 1 and 6 h of treatment, respectively.

### Nucleotide Variation, Population Structure, and Haplotype Analysis of *HvmTERF* Genes

To reveal the variation landscape of *HvmTERF*s, public resequencing data of barley were employed to detect *HvmTERF*-related SNPs. The SNP calling pipeline yielded a total of 481 *HvmTERF*-related SNPs or approximately 8.01 SNPs per gene, representing the most comprehensive variation dataset of *HvmTERF*s to date (Supplementary Table 12). The majority of *HvmTERF*-related SNPs (70.68%) were located in the genic region, including 159 synonymous, 133 missense, 44 intron, 3 splice region, and 1 stop-gain variant (*HvmTERF*42) (Supplementary Table 13). The overall transition/transversion (Ts/Tv) ratio was 2.317, with A/G (35.55%) and C/T (34.30%) ranking as the most popular allelic substitution patterns. These results indicated that there was fewer purine to purine or pyrimidine to pyrimidine mutation than there was pyrimidine to purine or purine to pyrimidine mutations.

**Figure 8:**
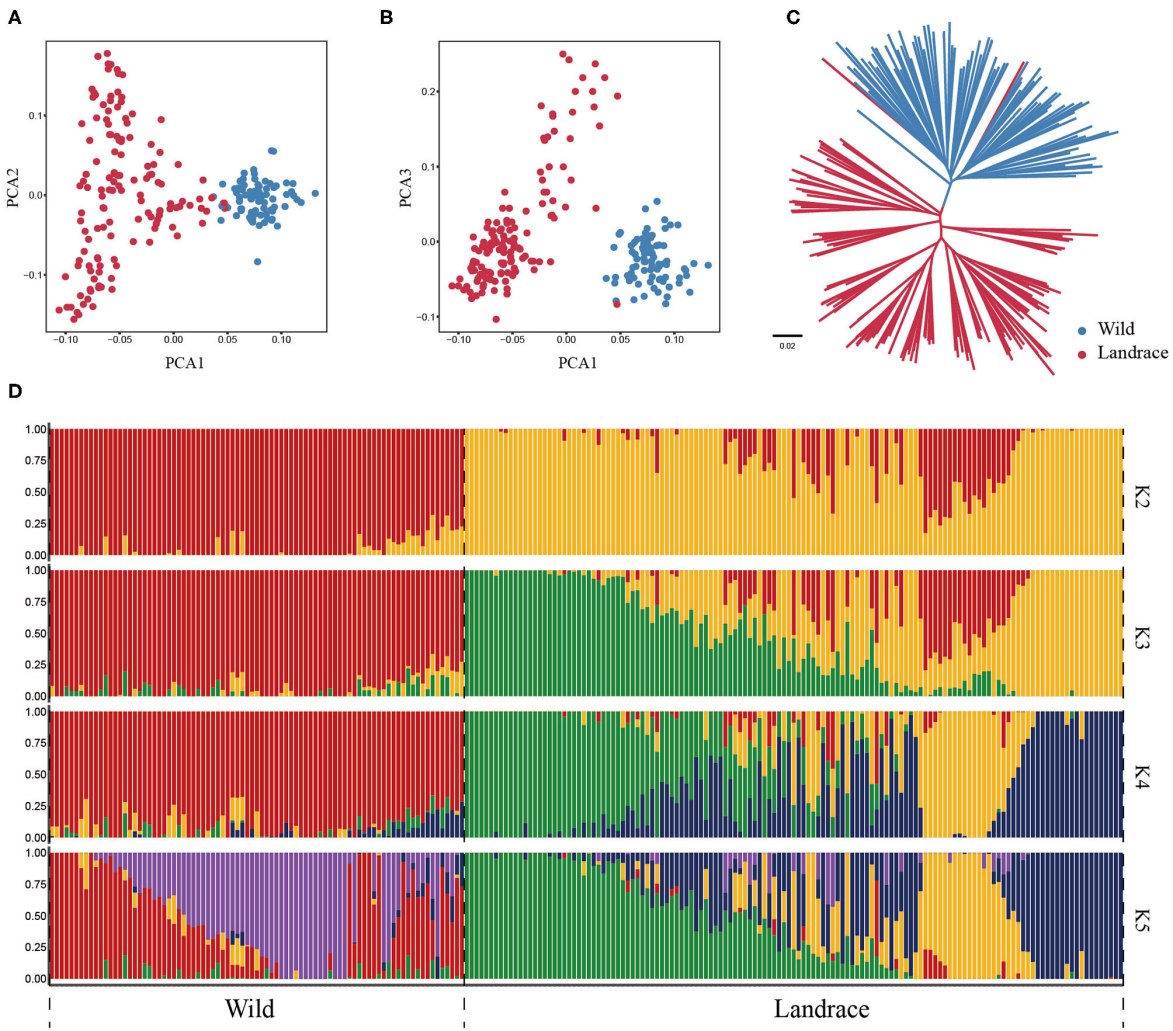
Population structure of wild barley accessions and landraces based on *HvmTERF*-related SNPs. **(A)** Principal component analysis PC1 vs. PC2, **(B)** Principal component analysis PC1 vs. PC3, **(C)** The NJ phylogenetic tree, **(D)** Population structure with K ranging from 2 to 5.

To further investigate the genetic relationship between wild and landrace barley populations, the PCA was conducted using *HvmTERF*-related SNPs. The first eigenvector explained 23.11% of the total genetic variance and captured the biological differentiation that separated wild barley from landrace barley. The second and third eigenvectors explained 12.16 and 11.10% of the variance, respectively, and distinguished the accessions geographically (Figures 8A,B; Supplementary Table 14). The same population affinities were recovered in the phylogenetic tree with a precise accession relationship (Figure 8C). ADMIXTURE analysis also recapitulated these findings (Figure 8D). When *K* = 2, a clear separation was observed in accordance with biological differentiation between wild and landrace barley. As *K* increased to 5, a definite separation was presented in accordance with the geographical source. Remarkably, a certain proportion of genetic admixture between wild and landrace barley was observed, suggesting the potential domestication origin of cultivated barley and ongoing gene flow between wild and landrace barley.

Population-based nucleotide diversity (π) was estimated based on *HvmTERF*-related SNPs. The nucleotide diversity decreased only 4.5% from wild barley (0.2491) to landrace barley (0.2380), indicating that this gene family suffered a weak genetic bottleneck in the process of domestication (Supplementary Figure 11). Wright's F-statistic is an informative indicator that measures population differentiation and gene flow intensity (Wright, 1951). Populations with Fst values >0.25 are considered highly differentiated (Fong et al., 2016). A relatively low Fst index (0.1310) was obtained between wild and landrace barley in accordance with the *HvmTERF*-related SNPs, indicating that this gene family was not subjected to strong selective pressure during barley domestication.

**Figure 9:**
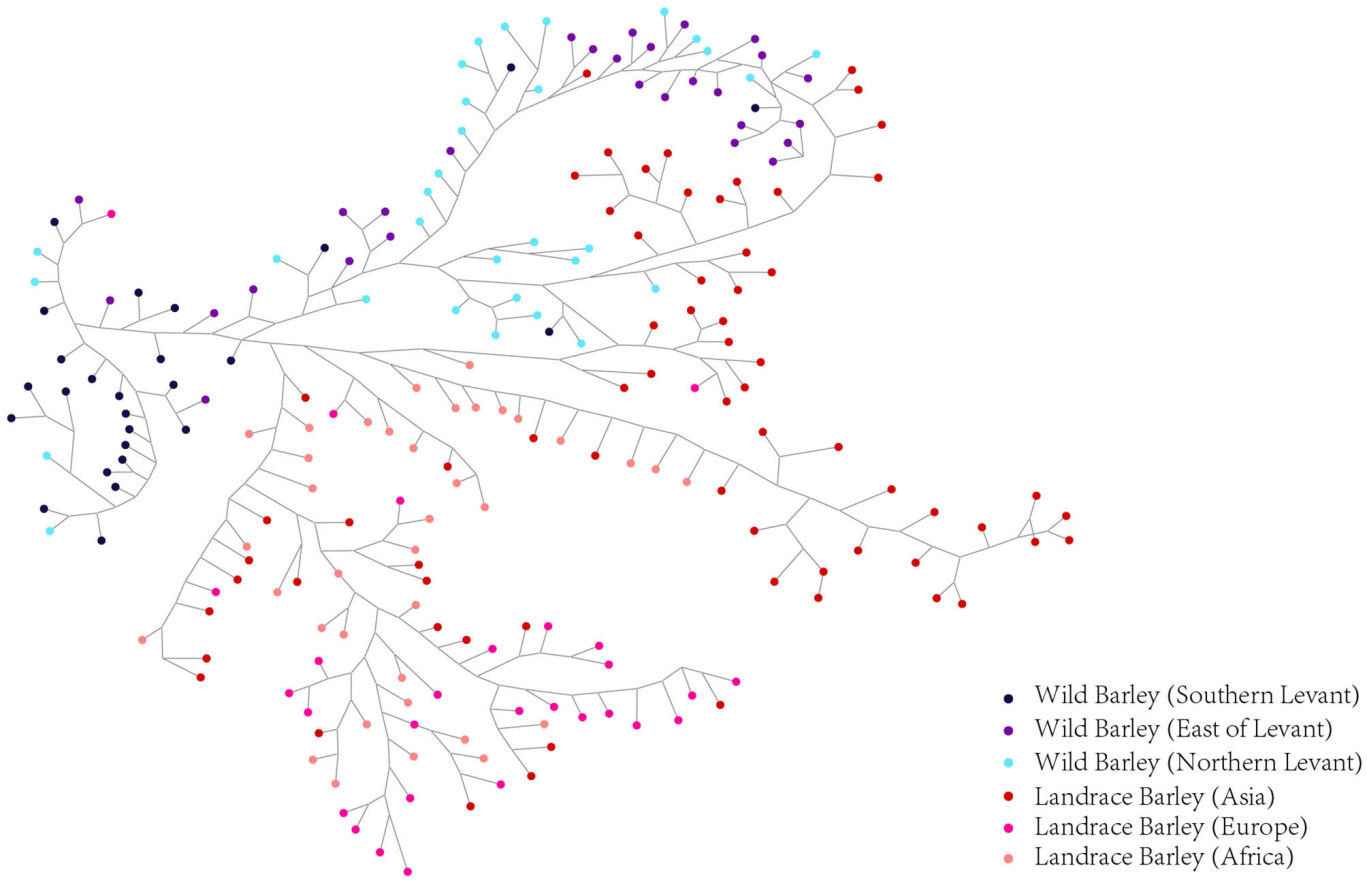
Median-Joining network analysis of wild barley accessions and landraces based on *HvmTERF*-related SNPs. Wild barley accessions were divided into Southern Levant, Northern Levant, and East of Levant groups. Landraces were divided into Asia, Europe, and Africa groups.

Haplotype dissection and comparison provide invaluable resources for understanding the evolutionary and domestication processes of important traits (Jan et al., 2019). To acquire a more precise depiction of the haplotype network, we constructed the complete haplotypes for the 220 accessions using *HvmTERF*-related SNPs. The median-joining method network generated a total of 481 *HvmTERF* haplotypes (one haplotype per accession) consisting of distinct wild and landrace groups (Figure 9). No shared haplotype between wild and landrace barley was observed in the network. The highly diverse wild accessions displayed geographical clustering patterns in terms of the Southern Levant (such as Israel and Jordan), Northern Levant (such as Syria and Turkey), and East of Levant (such as Iraq, Iran, and Central Asian counties). For landrace haplotypes, a geographical clustering pattern was obtained. However, a certain portion of accessions displayed no geographical clustering of haplotypes; for example, many shared haplotypes from Central Asia and Europe were observed in the median-joining network, suggesting a complex domestication process of *HvmTERF* in barley.

## Discussion

### Characterization of the *mTERF* Gene Family in Barley

The mTERF family, firstly identified in vertebrate mitochondria, is implicated in the regulation of organellar transcription, translation, and DNA replication (Quesada, 2016; Xiong et al., 2020). With the accomplishment of various whole genome sequencing projects, the identification and characterization of *mTERF* genes have been widely conducted in diverse plant species (Tang et al., 2019). As an inbreeding, diploid and temperate cereal crop, barley is well-studied in terms of cytology, genetics and molecular biology. However, its large genome size and high transposon content have long hindered barley genome assembly projects (Schulte et al., 2009). In recent years, the first physical sequence assembly for barley and its subsequent chromosome-scale reference sequence assembly (Morex V1), as well as the improved annotated reference genome assembly (Morex V2), have formed the basis for the identification and characterization of related gene families (Jayakodi et al., 2020). In this study, we carried out a comprehensive search for putative *HvmTERF*s, and a total of 60 *HvmTERF*s were identified in the barley Morex V2 genome assembly. Compared with its previous versions, the numbers of identified *HvmTERF*s were only 40 and 51 for the barley physical sequence assembly and Morex V1 assembly, respectively (Supplementary Table 15). In addition, in the Morex V1 assembly, two *HvmTERF* members named HORVU0Hr1G013680 and HORVU0Hr1G017090 were not mapped to the reference genome. However, they were anchored to chromosome 5 (HORVU.MOREX.r2.5HG0420050, *HvmTERF*20) and chromosome 6 (HORVU.MOREX.r2.6HG0509710, *HvmTERF*49) in the Morex V2 assembly. Each of the *HvmTERF*s contains the notable conserved mTERF domain. No premature termination codon was found in the coding region of *HvmTERF* genes, and most of them were supported by barley ESTs, ensuring the authenticity of gene family identification. Thus, this is the first gene family identification using the Morex V2 assembly at the whole genome-wide level, and the most updated and comprehensive information on the *mTERF* gene family in barley has been obtained to date.

The improved genome assembly also provided the definite physical locations of *HvmTERF*s. The *HvmTERF*s were distributed unevenly across seven chromosomes. The maximum number of *HvmTERF*s was located on the long arm end of chromosome 6, with a total of 19 *HvmTERF*s. Most of the *HvmTERFs* were located at the distal ends of the chromosome, but they were absent from the short arm of chromosome 3 and the long arm of chromosome 5. Similar findings were reported in other barley gene families, such as the non-specific lipid transfer protein (LTP) gene family and auxin/indole-3-acetic acid (*Aux/IAA*) gene family (Zhang et al., 2019; Shi et al., 2020). A possible cause might be that the distal and proximal ends of chromosomes are more gene-rich than the middle regions of chromosomes in barley (Mayer et al., 2012). In cereals, meiotic homologous chromosome recombination is skewed toward the distal regions of the chromosomes, leading to the biased distribution of genes that are concentrated in the distal regions (Higgins et al., 2012). The uneven distribution of recombination-rich regions ensures that there is abundant genetic diversity available to respond to various stressful conditions and dynamic environmental changes (Zhang et al., 2019).

In metazoans, the *mTERF* gene family contains four to five members (Roberti et al., 2009). In contrast, the *mTERF* gene family has expanded to approximately 30 members in land plants (Quesada, 2016). For example, there are 35 *mTERF*s in *Arabidopsis*, 33 in rice, 31 in maize, and 25 in grape. In this study, a total of 60 *HvmTERF* genes were identified in the barley reference genome, approximately two times as many as other higher plants. Given the complicated regulation of organellar genome transcription in land plants, the expansion of the *mTERF* gene family in barley could be induced by a complex mechanism. To gain insights into the evolutionary relationship within the *mTERF* gene family, we first constructed a phylogenetic tree using the mTERF proteins from barley, rice, and *Arabidopsis*. The mTERF proteins were categorized into nine subfamilies based on the classification set forth by Zhao (Zhao et al., 2014). Within the same subfamily, the gene structure and protein motif organization were highly conserved, supporting the phylogenetic analysis, and classification results. The phylogenetic tree further showed that eight subfamilies (subfamilies I–VIII) contained the mTERF proteins from these three species (barley, rice, and *Arabidopsis*), suggesting that these mTERF proteins evolved from common ancestors and then expanded independently in each species. Most of the subfamilies possessed comparable numbers of mTERF proteins, whereas monocot-specific subfamily IX does not contain any mTERF proteins from *Arabidopsis*, suggesting that subfamily IX formed after the divergence of monocots and dicots (Zhao et al., 2014). Furthermore, only 13 rice mTERF proteins were clustered in Group IX. In contrast, 39 mTERF proteins, more than half of the total mTERFs in barley, were assigned to this subfamily. Thus, we speculated that *HvmTERF*s within subfamily IX may have experienced noticeable expansion.

Gene duplication contributes to the expansion and evolution of gene families (Shi et al., 2020). To reveal the expansion mechanism of *HvmTERF*s, segmental and tandem duplication events were investigated. Fourteen pairs of *HvmTERF*s underwent gene duplication, including seven segmental and seven tandem duplication events. Remarkably, 17 *mTERF* genes consisting of 13 duplicated pairs contributed to the expansion of subfamily IX. Collectively, both segmental and tandem duplication contributed to the expansion of the *HvmTERF* gene family, mainly in subfamily IX, and further led to twice as many *mTERF* genes in barley as in other species. Moreover, the Ka/Ks values of all the paralogous gene pairs were <1, suggesting that they all underwent purifying selection.

### *HvmTERF* Genes May Play Important Roles During Plant Growth, Abiotic Stress, and Phytohormone Responses

To obtain preliminary insight into the biological function of *mTERF*s, we checked the *cis*-elements in the promoter regions of *HvmTERF*s. The promoter regions contained various *cis*-elements associated with development/tissue specificity, promoter/enhancer elements, light responses, circadian control rhythms, and external stimuli and hormone responses, suggesting that *HvmTERF*s are involved in multiple biological processes. Since *cis*-element prediction was carried out based on a bioinformatics approach, further experimental validation is also required.

In vertebrates, the biological function of *mTERF*s in the regulation of mitochondrial transcription, replication, and translation has been well-documented (Castillo et al., 2019). Although land plants possess more *mTERF*s than mammals, the functions of *mTERF*s in plants are rather limited (Kleine and Leister, 2015; Quesada, 2016). Based on their loss-of-function phenotypes, which have mainly been characterized in *Arabidopsis* and maize, *mTERF*s in land plants are required for OGE and play essential roles in plant growth and development (Ding et al., 2019). In this study, the specific spatiotemporal expression of *HvmTERFs* in different developmental stages and tissues suggested that *HvmTERF*s might potentially play a vital role in various plant growth and developmental processes. *HvmTERF2, HvmTERF16*, and *HvmTERF58* (orthologous to *RUGOSA2, SL1/mTERF3*, and *mTERF6* in *Arabidopsis*, respectively) displayed high expression levels in the studied tissues and stages. In *Arabidopsis*, these orthologous genes are essential for plant photosynthesis, mitochondrial, and chloroplastic gene expression and development, and leaf patterning and organogenesis (Quesada et al., 2011; Jiang et al., 2020). *HvmTERF24* was the most highly expressed gene in different organs. However, there is rather limited information on the functions of its orthologous gene in *Arabidopsis* (*AtmTERF12*). Recent research has only demonstrated that *AtmTERF12* is not involved in the response to salt stress (Xu et al., 2017). Several tissue- and stage-specific genes were also identified. For instance, *HvmTERF14* showed preferential expression in young inflorescences, whereas its ortholog *mTERF15* is required for the *cis*-splicing of mitochondrial *nad2* intron 3 in both *Arabidopsis* and maize and further regulates the small kernel phenotype in maize, implying that *HvmTERF14* may achieve different functions in barley compared with other species (Hsu et al., 2014; Yang et al., 2020). Homologous analysis might provide information on the role of *HvmTERFs*. However, to ascertain *HvmTERF* function still requires further detailed and extensive experimental work (Yin et al., 2021).

In contrast to animals, plants are sessile organisms that are continuously exposed to and cannot escape environmental stresses. During evolution, the expansion and diversification of gene families played important roles in plant adaptation or tolerance to environmental extremes (Xu et al., 2017). A wide range of mechanisms have evolved in plants to cope with adverse environmental stresses at the molecular level. Compared with the control, a total of three and four upregulated genes were identified under cold and salt treatment, whereas under metal poisoning stresses, a total of four zinc, five copper, and two cadmium toxicity stress-related *HvmTERF*s were identified. Since similar studies are rather scarce, further experimental validation is required. Therefore, the expression patterns of *HvmTERF*s in response to various stresses were further investigated by qRT-PCR. Most of the qRT-PCR results were consistent with the RNA-seq results. For example, both the RNA-seq and qRT-PCR results demonstrated that *HvmTERFs* 19, 23, and 58 were downregulated in response to salt stress. By contrast, several upregulated *HvmTERFs* were also detected in response to various stresses. For instance, *HvmTERF*21 was upregulated under salt and cold stress based on RNA-seq, while this gene was significantly upregulated by cold, salt, drought and ABA stress via qRT-PCR analysis. Homology analysis revealed the involvement of its orthologous gene *AtmTERF10* in salt tolerance, possibly through an ABA-mediated mechanism (Xu et al., 2017). Nonetheless, certain inconsistent expression patterns were also observed. RNA-seq data revealed that *HvmTERF*7 and *HvmTERF*46 were not induced by salt stress at the three root zones, while qRT-PCR analysis showed that these genes were significantly downregulated at 1, 3, 12, and 24 h and significantly upregulated at 6 h under salt treatment. Previous studies reported that *mTERF9* (orthologous to *HvmTERF7*) and *MAD1/mTERF5* (orthologous to *HvmTERF46*) contributed to salt tolerance in *Arabidopsis* (Robles et al., 2012, 2015; Núñez-Delegido et al., 2020). The inconsistent results between RNA-seq and qRT-PCR may be due to several putative reasons. First, the different barley varieties were used in the two experiments. The barley cultivar Clipper was used in RNA-seq, whereas the cultivar Morex was the experimental materials in qRT-PCR. Second, the plant materials were not exactly the same. The materials for qRT-PCR were roots, whereas the materials for RNA-seq were both roots and leaves. Third, the expression level of qRT-PCR was calculated based on the 2^−Δ*ΔCT*^ method, and the expression level of RNA-seq was estimated by FPKM. These two different calculation methods could not ensure that all the results are consistent. In brief, these results provide candidates for further functional investigation of *mTERF* genes in barley as well as in other cereal crops.

In addition, there was no correlation between expression patterns and phylogenetic relationships. The fate of *HvmTERF* genes from the same gene family could be described as neofunctionalization during expansion. For example, in subfamily IV, *HvmTERFs 2, 8*, and *46* showed relatively high expression in various tissues and developmental stages, whereas *HvmTERF20* exhibited preferential expression in developing inflorescences and senescing leaves, and *HvmTERF51* was not expressed in most of the developmental stages and tissues. Highly diverse expression patterns were also observed in subfamily IX, the most expanded subfamily. In addition, a divergent expression profile was investigated even for duplicated gene pairs. The duplicated genes *HvmTERF9* and *34* showed divergent spatiotemporal expression patterns. *HvmTERF34* was induced by copper, whereas its paralog, *HvmTERF9*, was significantly upregulated in the root meristematic zone under salt treatment. Similar patterns were also observed in other phylogenetic groups. These results suggested that close phylogenetic relationships are not essential for similar expression profiles, which was consistent with previous reports on other barley gene families (Li et al., 2019).

### Nucleotide Diversity Analysis Indicated That *HvmTERF* Genes Experienced a Weak Bottleneck During Barley Domestication

Domestication is a plant-animal coevolution process that occurs when wild species are exposed to new selective environments associated with human cultivation and use, leading to morphological and physical changes that distinguish domesticated taxa from their wild ancestors (Purugganan and Fuller, 2009; Purugganan, 2019). Cultivated barley, domesticated from its progenitor wild barley (*Hordeum vulgare* ssp. *spontaneum*), has experienced a series of genetic changes that have caused differences in plant architecture and growth habits, collectively called the domestication syndrome (Hammer, 1984; Doebley et al., 2006). Domestication of barley resulted in a concomitant bottleneck that reduced nucleotide diversity in alleles (Haas et al., 2020). However, little is known about the changes in *mTERF*s resulting from domestication in barley. In this study, 481 SNPs were identified from 60 *HvmTERF* genes across 220 wild and landrace barley accessions. The SNPs were distributed unevenly along the genomic sequence, including a total of 292 exon and 44 intron variations, which was consistent with a previous study showing higher polymorphism of SNPs in exon regions than in intron regions (Lu et al., 2019) but opposite to observations in other studies (Uçarli et al., 2016; Xia et al., 2017). The PCA, admixture, and phylogenetic analyses clearly divided all the accessions into landraces and wild barley accessions and further distinguished them geographically. We further examined the geographical distribution of these haplotypes and found that the wild barley populations from the Northern Levant and East of Levant regions appeared to contribute most directly to the genetic composition of Asian landraces, while Southern Levant barley populations made a great contribution to African and European landraces. The genetic constitution of barley landraces indicated multiple origins from wild progenitor populations that resulted in the initial domestication and subsequent migration of early agriculturalists along the axes of the Afro-Eurasian world (Poets et al., 2015). Although multiple wild populations provided the basis for the genetic constitution of landraces, the broad geographic range of landraces also showed various regional correlations.

Domestication also resulted in a concomitant bottleneck that reduced sequence diversity across many genes (Haas et al., 2020). The nucleotide diversity of wild accessions was relatively higher than that of landrace accessions, with a total decrease of ~4.5%. Compared with the previous study, the average reduction in nucleotide diversity was 27% from wild barley accessions to landraces across the genome (Russell et al., 2016). The significantly lower nucleotide diversity loss passing from wild barley accessions to landraces in this study indicated that the *HvmTERF* gene family might have suffered simple bottleneck effects, rather than selection, in the process of barley domestication. This result was also verified by the relatively low Fst index. No significant divergence occurred between wild barley accessions and landraces regarding *HvmTERF*s. One plausible reason is that the hitchhiking effect reduced the nucleotide diversity of the linked loci associated with domestication (Kilian et al., 2006).

As in other plants, *mTERF*s are characterized as evolutionarily conserved and fundamentally universal signaling pathways. However, the comprehensive characterization of barley *mTERF* gene family remains to be elucidated in detail. Our data on the physicochemical properties, phylogenetic relationships, gene structures, conserved motifs, expansion patterns, expression profiles, and genetic variations will provide essential clues for investigating the biological functions and evolutionary history of *mTERF* gene family in barley.

## Conclusion

In this study, a total of 60 *mTERF* genes were identified in barley, about two times as many as those in *Arabidopsis* and rice. Phylogenetic analysis categorized these genes into nine subfamilies, with approximately half of them assigned to subfamily IX, which was supported by exon-intron structure and conserved motif analyses. Both segmental and tandem duplications contributed significantly to the expansion of *HvmTERFs*, mainly in subfamily IX. *Cis*-acting regulatory element, expression profile and co-expression network analyses suggested that *HvmTERF*s might be involved in the development process, tolerance to diverse stresses and response to plant hormones. qRT-PCR analysis also revealed that *HvmTERF*21 and *HvmTERF*23 were significant induced by several abiotic stresses and/or phytohormone treatment, and these genes could be considered candidates for further functional studies. Finally, genetic variation analysis demonstrated that *HvmTERF*s may have experienced a weak genetic bottleneck, rather than selection, during the domestication process from wild to landrace barley. Taken together, our findings will not only provide a solid foundation for further evolutionary analysis but also facilitate the functional study of *HvmTERF*s and the molecular breeding of barley.

## Data Availability Statement

The original contributions presented in the study are included in the article/Supplementary Material, further inquiries can be directed to the corresponding author/s.

## Author Contributions

XW, FW, and LC designed and supervised the project. TL, WP, YY, and YLiu performed the data analysis. WP and YLiu performed the experiments. TL, YLi, and LC drafted the manuscript. All authors contributed to the article and approved the submitted version.

## Conflict of Interest

The authors declare that the research was conducted in the absence of any commercial or financial relationships that could be construed as a potential conflict of interest.

## Supplementary Material

Supplementary Figure 1 Schematic geographic distribution of 220 wild barley and landraces accessions from different regions.

Supplementary Figure 2 Sequence characteristics and conserved amino acids ratio of mTERF proteins in four species. **(A)** Barley, **(B)** Rice, **(C)** *Arabidopsis*, **(D)** Maize.

Supplementary Figure 3 Distribution of *HvmTERF* genes on barley chromosomes. Chromosome numbers are shown at the top of each chromosome. The scale (Mb) is indicated on the left.

Supplementary Figure 4 GO enrichment (biological process) of *HvmTERF* co-expressed genes in the WGCNA network.

Supplementary Figure 5 GO enrichment (cellular component) of *HvmTERF* co-expressed genes in the WGCNA network.

Supplementary Figure 6 GO enrichment (molecular function) of *HvmTERF* co-expressed genes in the WGCNA network.

Supplementary Figure 7 The expression analysis of *HvmTERF* genes in response to salt. Error bar represent the standard error of the mean. One asterisk (^*^) indicates 0.05 significance level. Double asterisk (^**^) indicates 0.01 significance level.

Supplementary Figure 8 The expression analysis of *HvmTERF* genes in response to drought. Error bar represent the standard error of the mean. One asterisk (^*^) indicates 0.05 significance level. Double asterisk (^**^) indicates 0.01 significance level.

Supplementary Figure 9 The expression analysis of *HvmTERF* genes in response to cold. Error bar represent the standard error of the mean. One asterisk (^*^) indicates 0.05 significance level. Double asterisk (^**^) indicates 0.01 significance level.

Supplementary Figure 10 The expression analysis of *HvmTERF* genes in response to ABA. Error bar represent the standard error of the mean. One asterisk (^*^) indicates 0.05 significance level. Double asterisk (^**^) indicates 0.01 significance level.

Supplementary Figure 11 Nucleotide diversity of wild barley and landraces accessions. The horizontal line inside the box corresponds to the median of this distribution, the bottom and top of the box are the first and third quartiles and data points outside the whiskers can be considered as outliers. The nucleotide diversity was calculated based on *HvmTERF*-related SNPs.

Supplementary Table 1 Accession number and samples information of RNA-seq data used in this study.

Supplementary Table 2 The primers used in the qRT-PCR.

Supplementary Table 3 Summary information of the 220 wild barley accessions and landraces.

Supplementary Table 4 Characteristic features of *mTERF* gene family in barley.

Supplementary Table 5 Verification of the mTERF domains using the NCBI-CDD, SMART, HMMER, and InterPro online tools.

Supplementary Table 6 Consensus motifs analysis of mTERF domains in different species.

Supplementary Table 7 The segmental and tandem duplication events of *HvmTERF*s.

Supplementary Table 8 The Ka/Ks ratios and estimated divergence time between barley and other five species (*A. thaliana, B. distachyon, O. sativa, Vitis vinifera, Zea mays*).

Supplementary Table 9 Characteristics of *cis*-acting regulatory elements in the promoter regions of the *HvmTERF* genes.

Supplementary Table 10 List of putative miRNAs targeted to *HvmTERF* genes identified by psRNATarget online tool.

Supplementary Table 11 GO enrichment of *HvmTERF* co-expressed genes.

Supplementary Table 12 The genetic variation information (VCF format) of *HvmTERF*-related SNPs across the 220 wild barley accessions and landraces.

Supplementary Table 13 Distribution of SNPs within various genomic regions.

Supplementary Table 14 Tracy-Widom (TW) statistics and *P*-values for the first 10 eigenvalues in the PCA of *HvmTERF*-related SNPs.

Supplementary Table 15 The number of *HvmTERF*s from different barley assemblies.

## Abbreviations

ABA: abscisic acid
ABRE: ABA-responsive elements
Aux/IAA: auxin/indole-3-acetic acid
BLAST: basic local alignment search tool
C3H: cysteine3histidine
CDD: conserved domains database
CDS: coding sequence
EST: expressed sequence tag
FDR: false discovery rate
FPKM: fragments per kilobase per million
ERE: estrogen response element
Fst: Wright's F-statistic
GCN4: general control non-repressible-4
GO: gene ontology
GRAVY: grand average of hydropathicity
GRF: growth-regulating factor
GSDS: Gene Structure Display Sever
GTF: Gene Transfer Format
HMM: hidden Markov model
HSP: heat shock protein
Ka: non-synonymous substitution rate
Ks: synonymous substitution rate
LTP: lipid transfer protein
LTR: long terminal repeat
MBS: myeloblastosis binding site
MDA1: mTERF DEFECTIVE IN Arabidopsis1
MeJA: methyl jasmonate
MEME: Multiple Em for Motif Elicitation
MSA-like: mitosis-specific activator
MW: molecular weight
MYB: myeloblastosis
NCBI: National Coalition Building Institute
NEP: nucleus-encoded RNA polymerase
NJ: neighbor-joining
OGE: organellar gene expression
PAML: Phylogenetic Analysis by Maximum Likelihood
PCA: principal component analysis
PEP: plastid-encoded RNA polymerase
pI: theoretical isoelectric point
qRT-PCR: Quantitative Real-time PCR
ROS: reactive oxygen species
SA: salicylic acid
SHOT1: SUPPRESSOR OF HOT1-4 1
SL1: SEEDLING LETHAL 1
SMART: simple modular architecture research tool
SNP: single nucleotide polymorphism
SOLDAT10: SINGLET OXYGEN-LINKED DEATH ACTIVATOR10
SRA: sequence read archive
Ts: transition
Tv: transversion
WGCNA: weighted gene co-expression network analysis.
