# Supplementary figures and images for: Identification, Characterization, and Expression Profile Analysis of the mTERF Gene Family and Its Role in the Response to Abiotic Stress in Barley (Hordeum vulgare L.)
Source: Front Plant Sci. 2021 Jul 15;12:684619. doi: 10.3389/fpls.2021.684619 (PMC8319850; doi:10.3389/fpls.2021.684619)

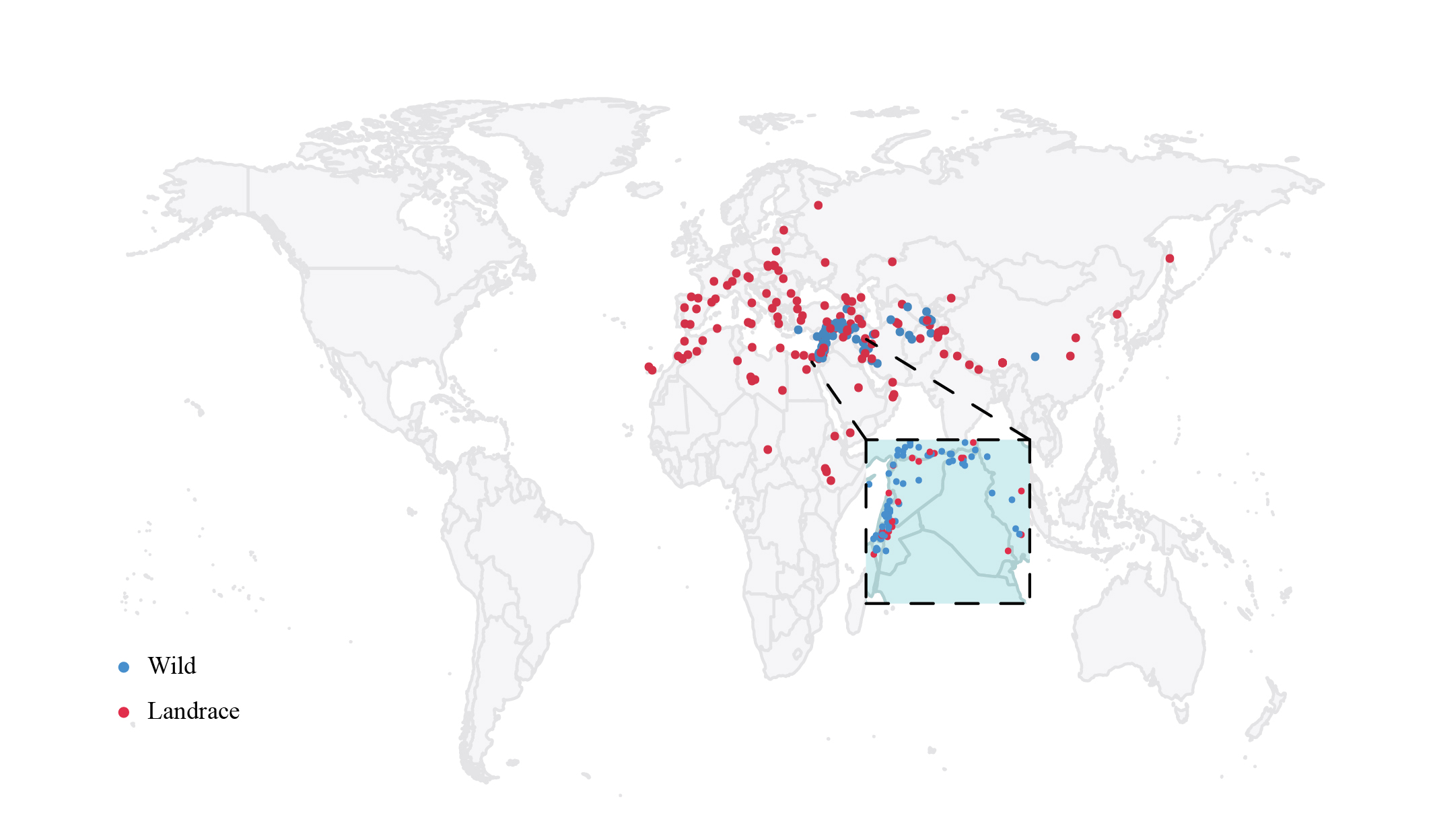

Supplement: Supplementary Figure 1 — Schematic geographic distribution of 220 wild barley and landraces accessions from different regions. [file Image_1.JPEG]

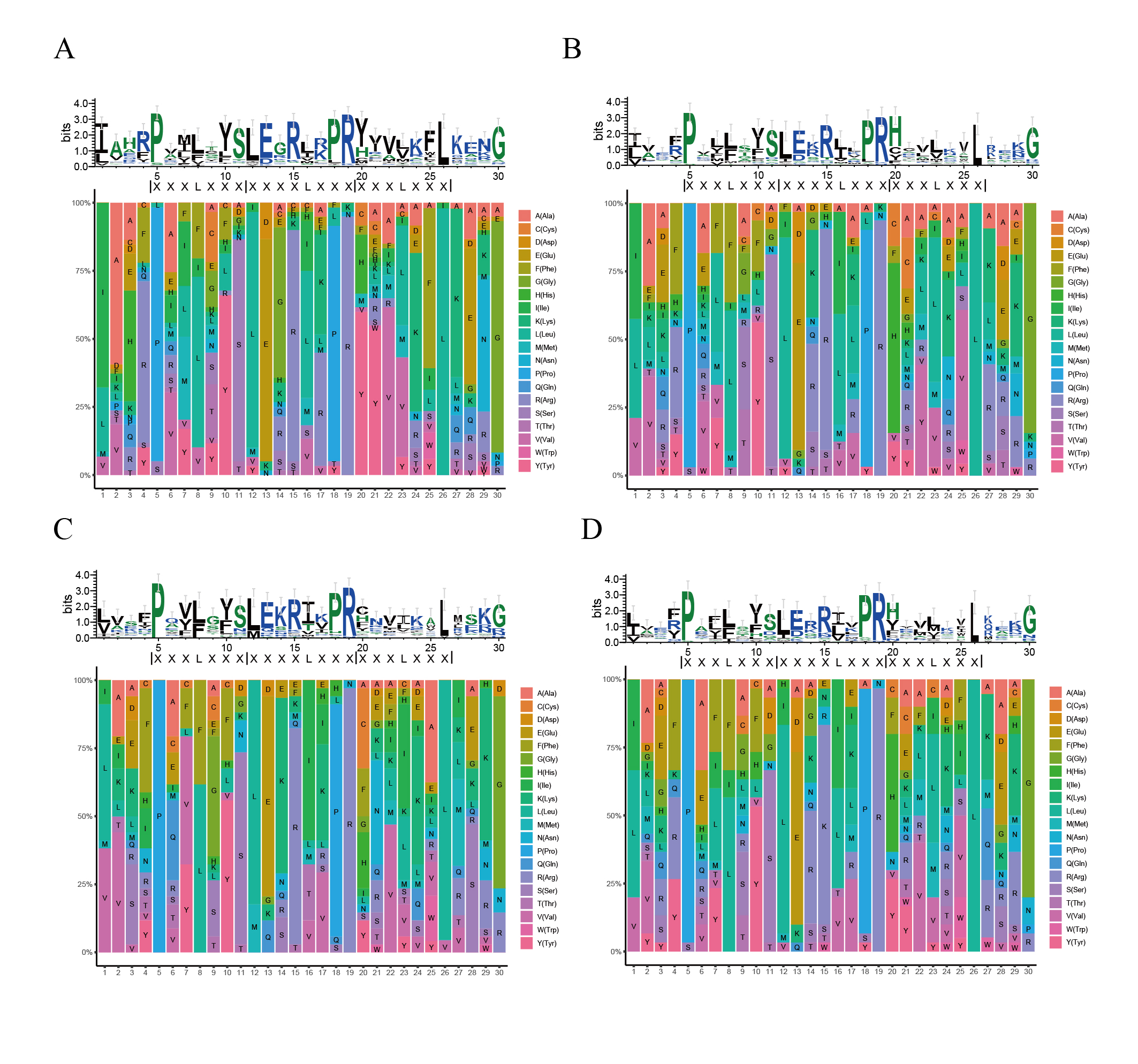

Supplement: Supplementary Figure 2 — Sequence characteristics and conserved amino acids ratio of mTERF proteins in four species. (A) Barley, (B) Rice, (C) Arabidopsis, (D) Maize. [file Image_2.JPEG]

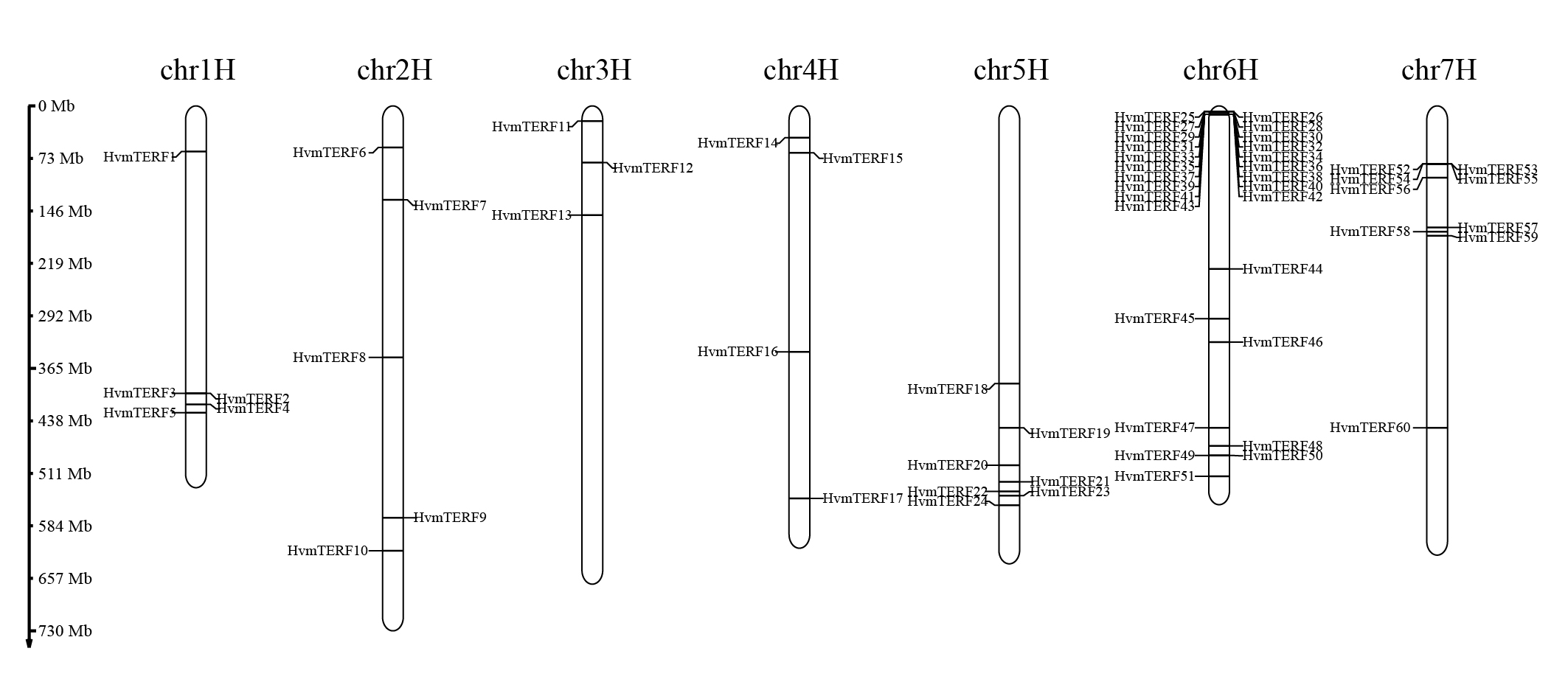

Supplement: Supplementary Figure 3 — Distribution of HvmTERF genes on barley chromosomes. Chromosome numbers are shown at the top of each chromosome. The scale (Mb) is indicated on the left. [file Image_3.JPEG]

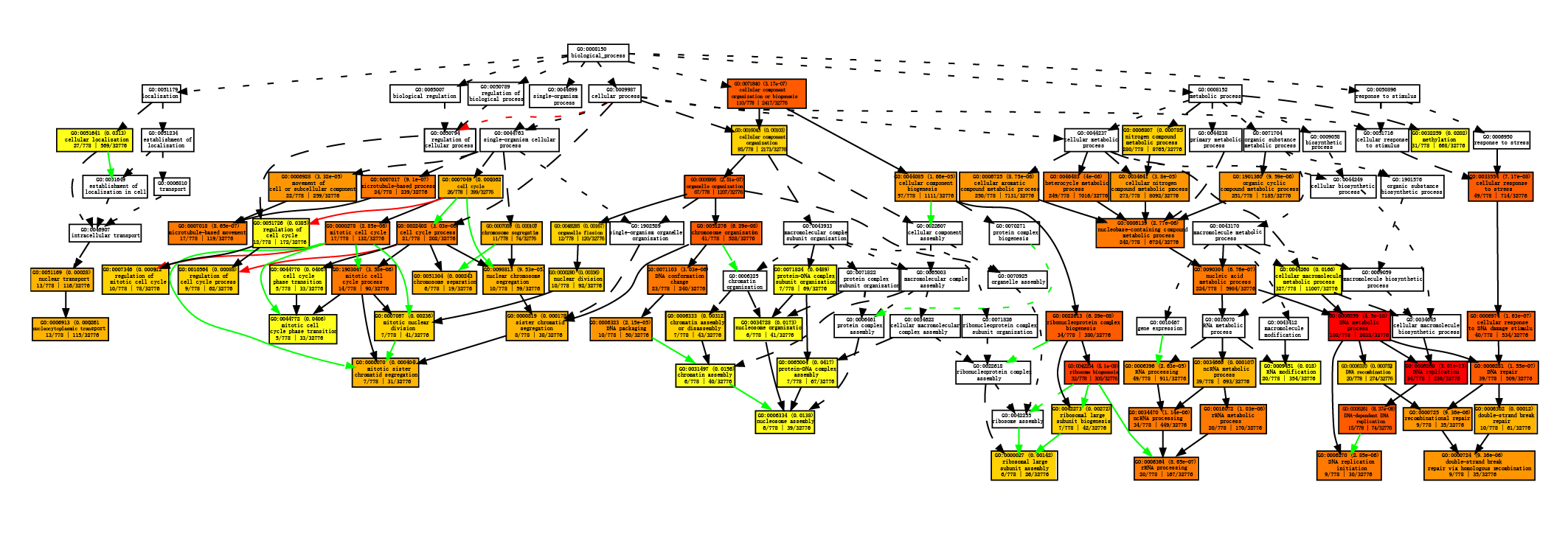

Supplement: Supplementary Figure 4 — GO enrichment (biological process) of HvmTERF co-expressed genes in the WGCNA network. [file Image_4.JPEG]

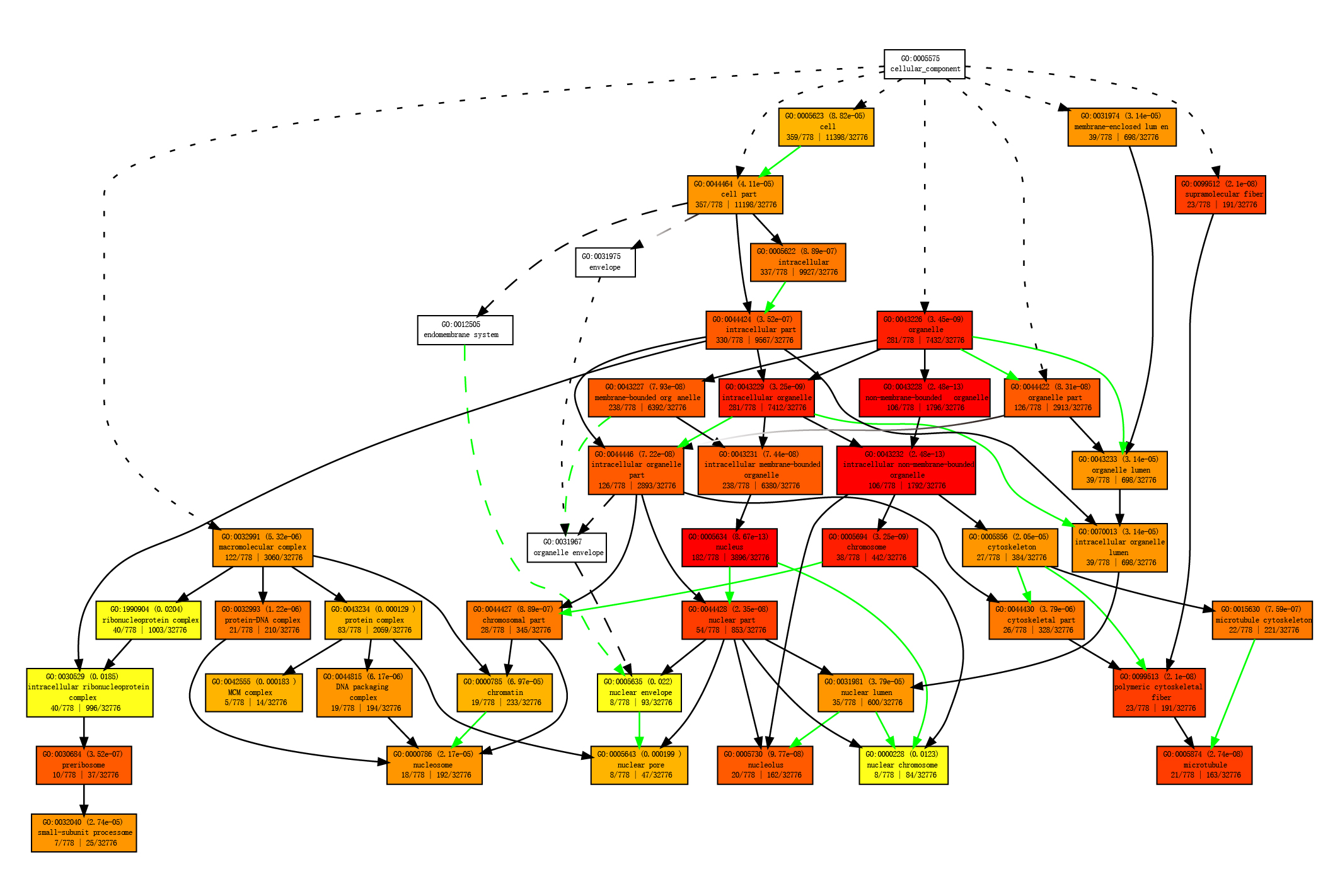

Supplement: Supplementary Figure 5 — GO enrichment (cellular component) of HvmTERF co-expressed genes in the WGCNA network. [file Image_5.JPEG]

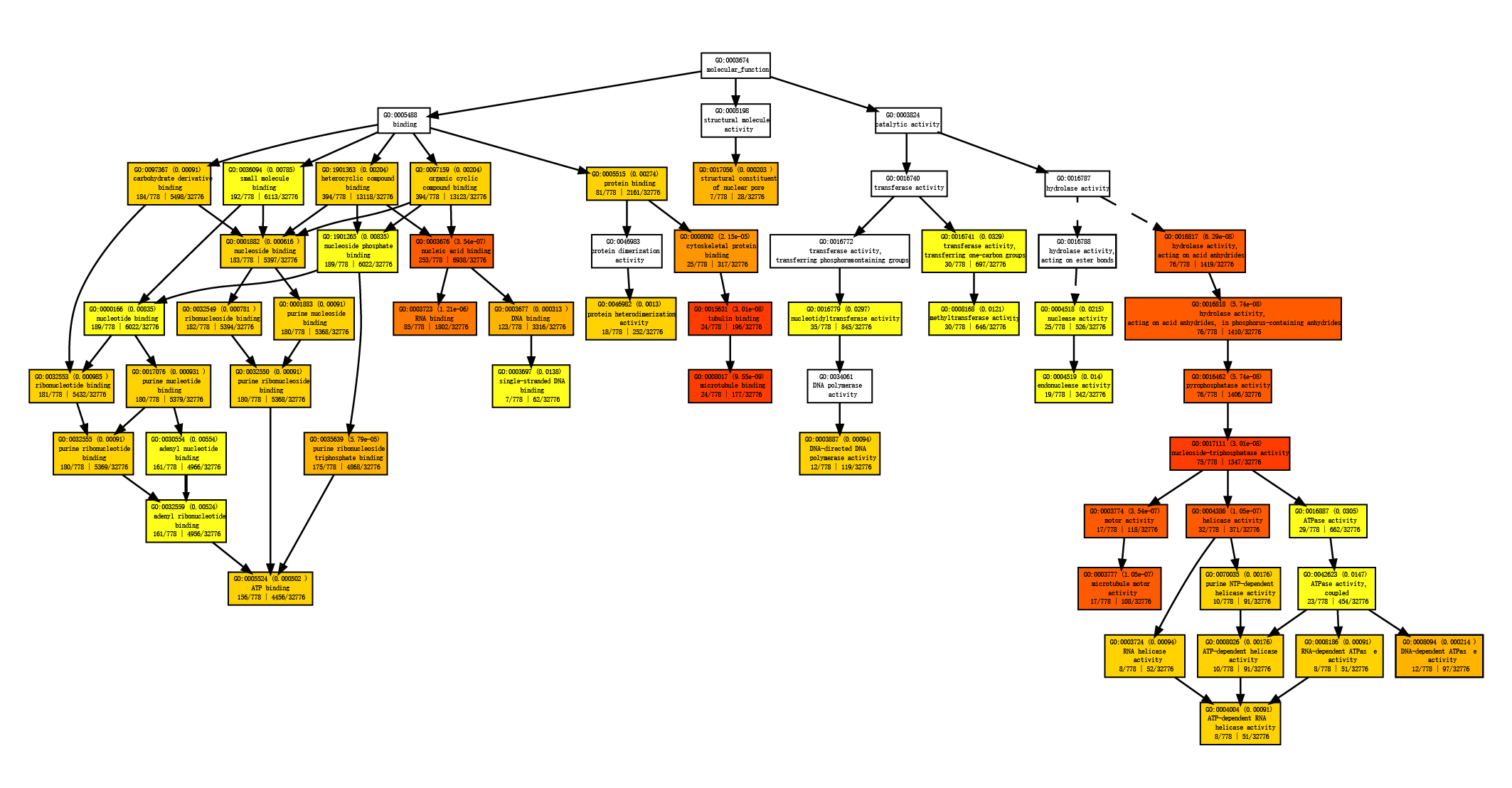

Supplement: Supplementary Figure 6 — GO enrichment (molecular function) of HvmTERF co-expressed genes in the WGCNA network. [file Image_6.JPEG]

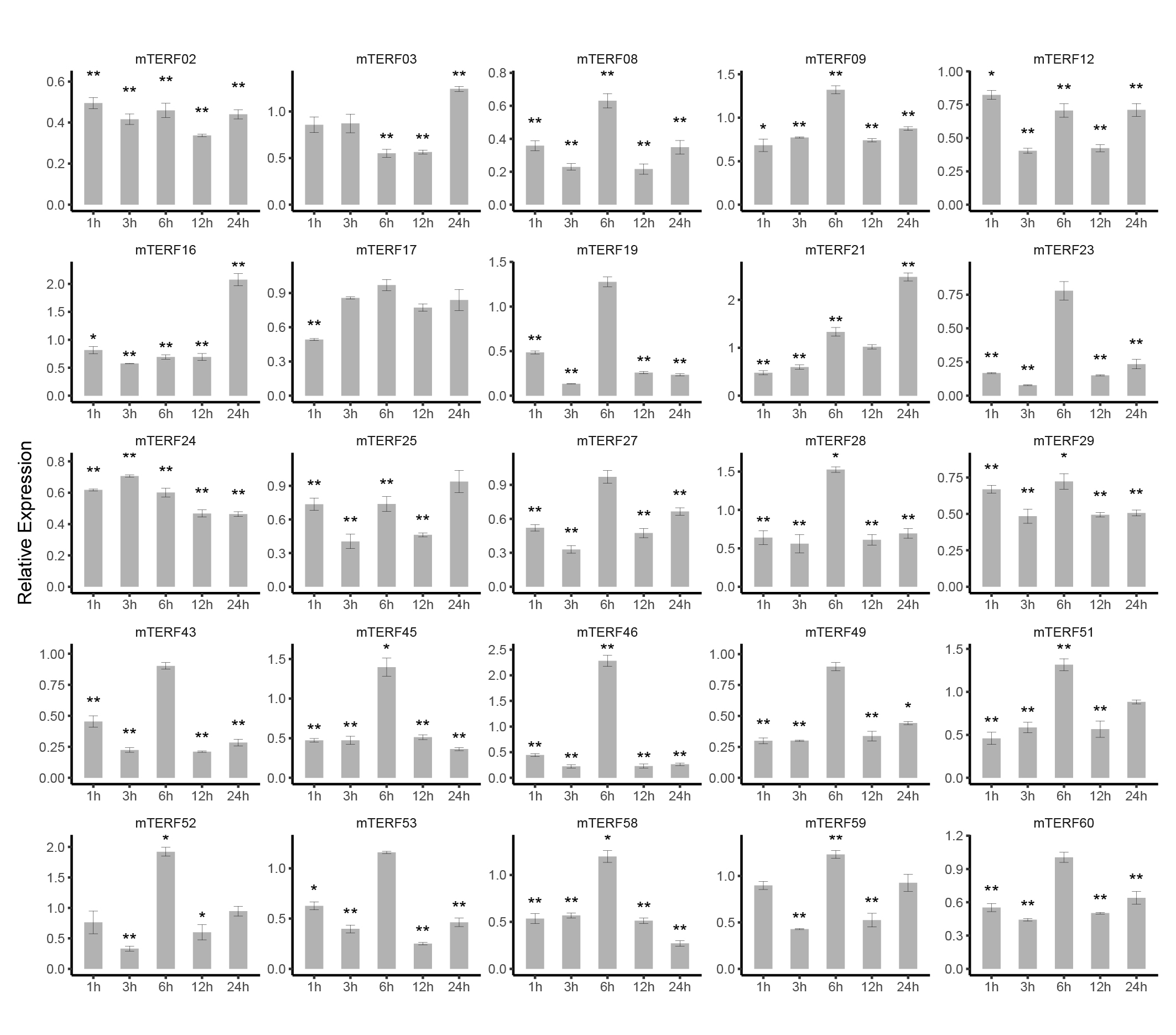

Supplement: Supplementary Figure 7 — The expression analysis of HvmTERF genes in response to salt. Error bar represent the standard error of the mean. One asterisk (*) indicates 0.05 significance level. Double asterisk (**) indicates 0.01 significance level. [file Image_7.JPEG]

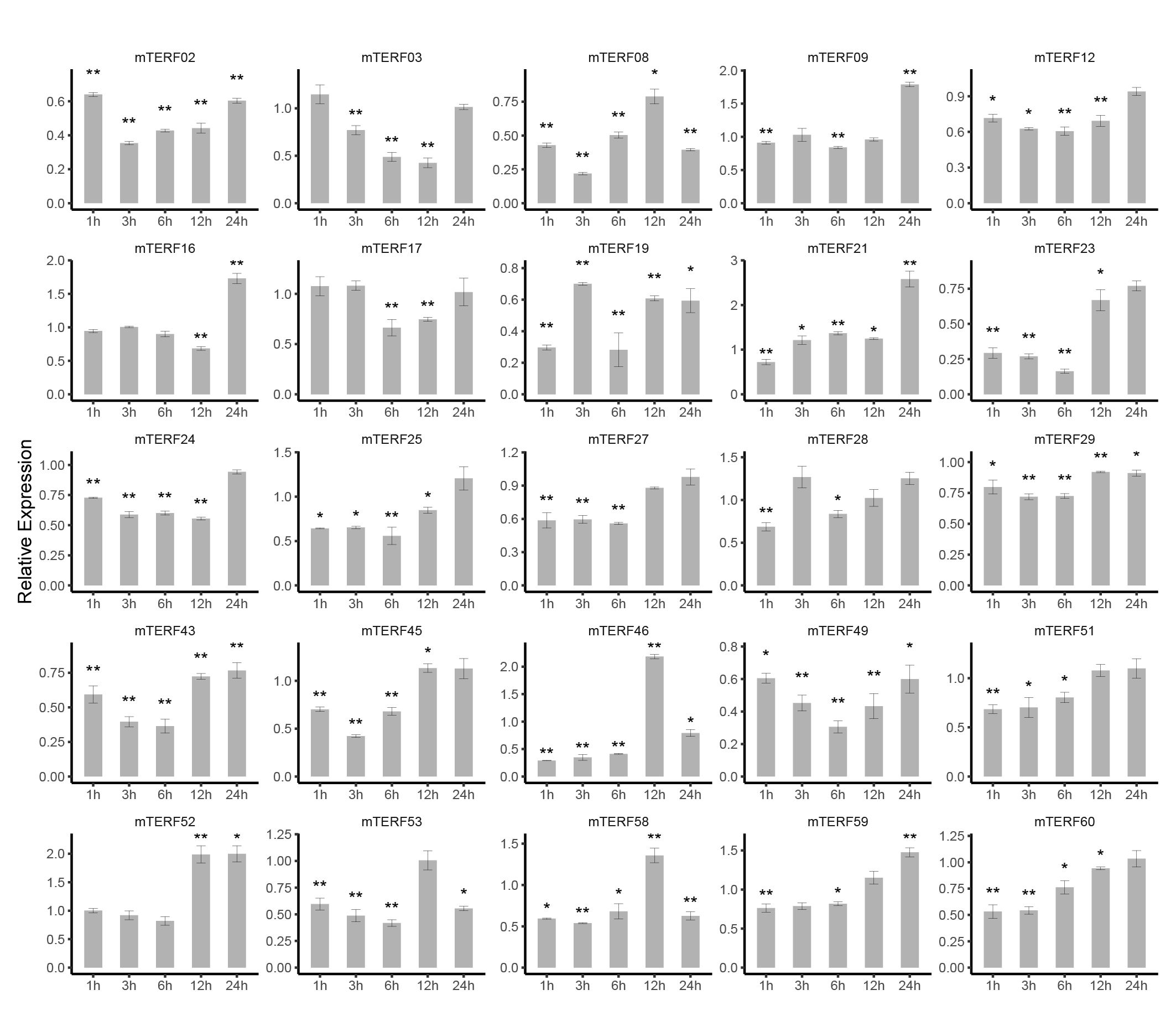

Supplement: Supplementary Figure 8 — The expression analysis of HvmTERF genes in response to drought. Error bar represent the standard error of the mean. One asterisk (*) indicates 0.05 significance level. Double asterisk (**) indicates 0.01 significance level. [file Image_8.JPEG]

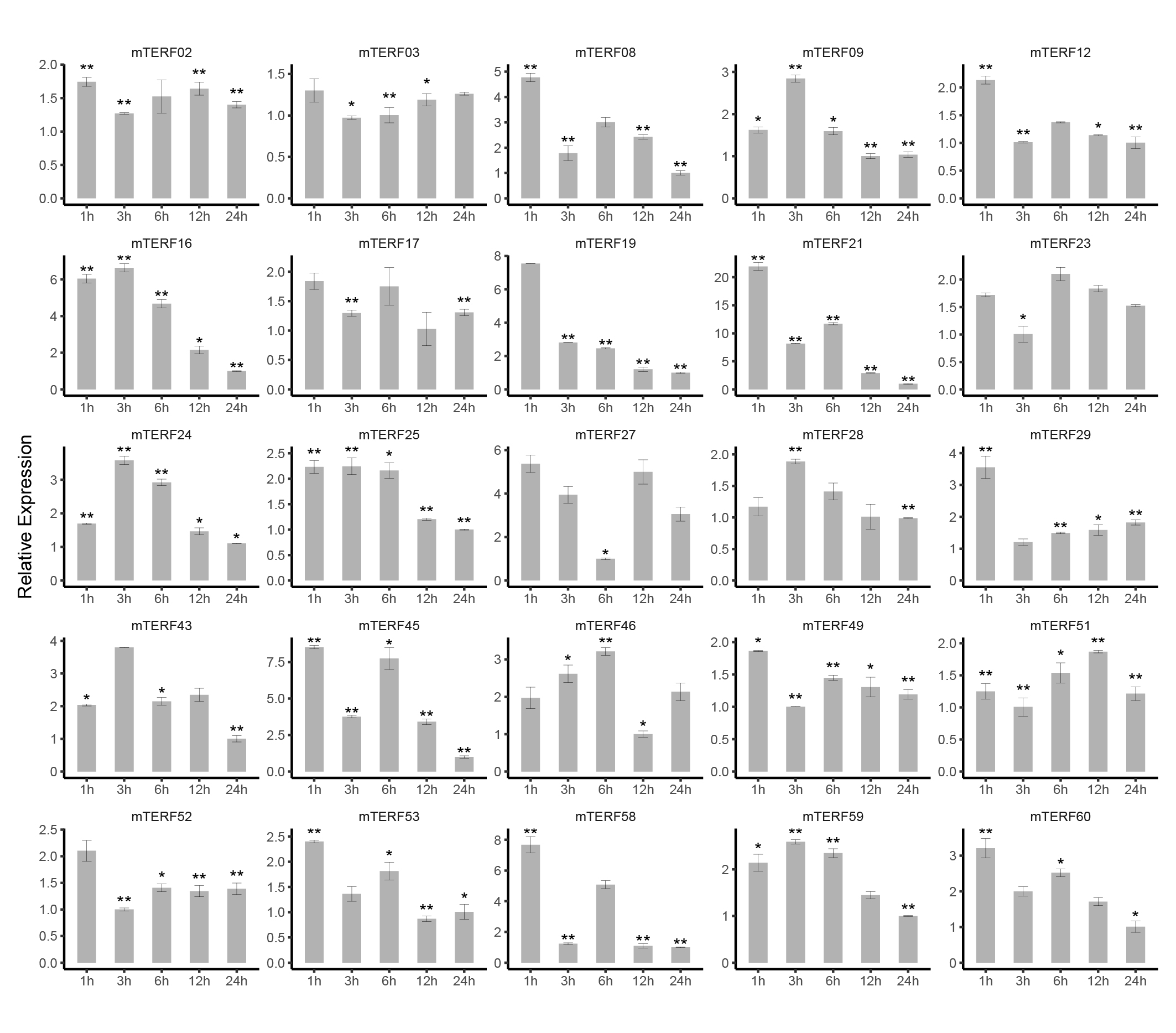

Supplement: Supplementary Figure 9 — The expression analysis of HvmTERF genes in response to cold. Error bar represent the standard error of the mean. One asterisk (*) indicates 0.05 significance level. Double asterisk (**) indicates 0.01 significance level. [file Image_9.JPEG]

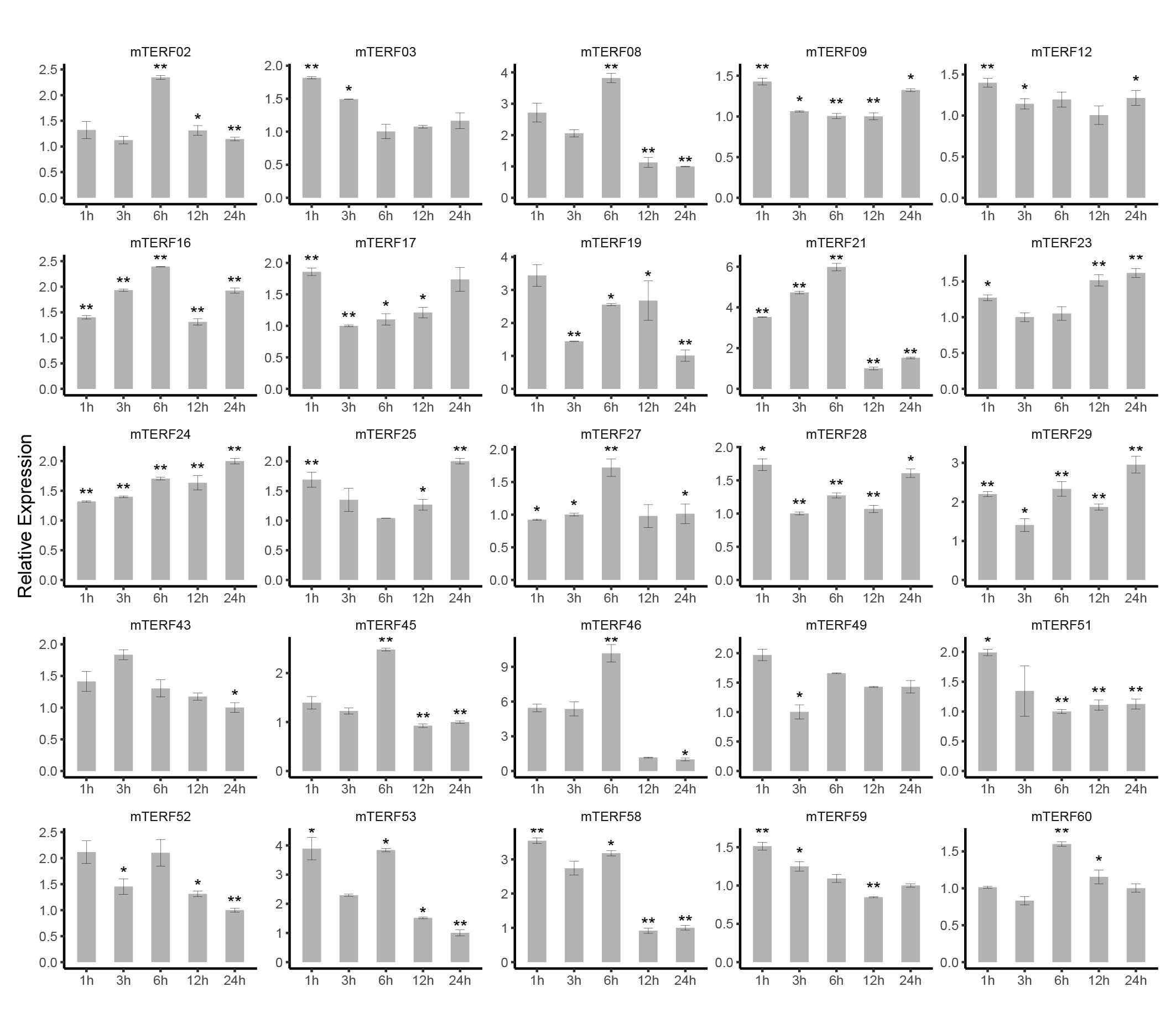

Supplement: Supplementary Figure 10 — The expression analysis of HvmTERF genes in response to ABA. Error bar represent the standard error of the mean. One asterisk (*) indicates 0.05 significance level. Double asterisk (**) indicates 0.01 significance level. [file Image_10.JPEG]

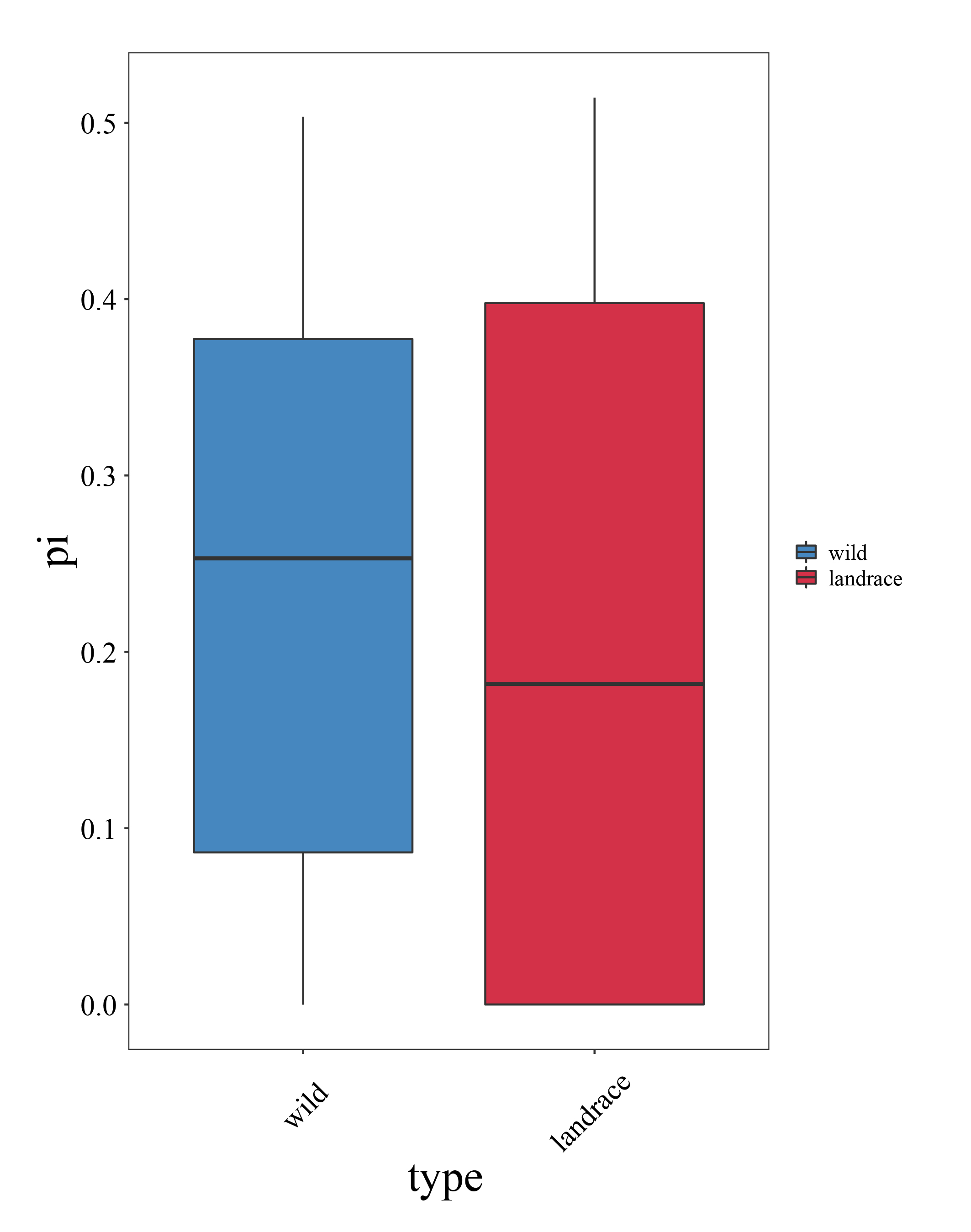

Supplement: Supplementary Figure 11 — Nucleotide diversity of wild barley and landraces accessions. The horizontal line inside the box corresponds to the median of this distribution, the bottom and top of the box are the first and third quartiles and data points outside the whiskers can be considered as outliers. The nucleotide diversity was calculated based on HvmTERF-related SNPs. [file Image_11.JPEG]
